# Comparative analysis of the nutritional, physicochemical, and bioactive characteristics of *Artemisia abyssinica* and *Artemisia arborescens* for the evaluation of their potential as ingredients in functional foods

**DOI:** 10.1002/fsn3.4431

**Published:** 2024-08-29

**Authors:** Qais Ali Al‐Maqtari, Norzila Othman, Jalaleldeen Khaleel Mohammed, Amer Ali Mahdi, Waleed Al‐Ansi, Abeer Essam Noman, Adel Ali Saeed Al‐Gheethi, Syazwani Mohd Asharuddin

**Affiliations:** ^1^ Micro‐Pollutant Research Centre (MPRC), Faculty of Civil Engineering and Built Environment Universiti Tun Hussein Onn Malaysia (UTHM) Batu Pahat Johor Malaysia; ^2^ Department of Food Science and Nutrition, Faculty of Agriculture, Food, and Environment Sana'a University Sana'a Yemen; ^3^ Department of Microbiology, Faculty of Science Sana'a University Sana'a Yemen; ^4^ Global Centre for Environmental Remediation (GCER) University of Newcastle and CRC for Contamination Assessment and Remediation of the Environment (CRC CARE) Newcastle New South Wales Australia

**Keywords:** *Artemisia abyssinica*, *Artemisia arborescens*, bioactive compounds, functional properties, nutritional value

## Abstract

*Artemisia abyssinica* and *Artemisia arborescens* are unique plants that show significant bioactive properties and are used for the treatment of a variety of diseases. This study assessed the nutritional values, functional properties, chemical composition, and bioactive attributes of these plants as functional nutritional supplements. Compared to *A. arborescens*, *A. abyssinica* had higher fat (4.76%), fiber (16.07%), total carbohydrates (55.87%), and energy (302.15 kcal/100 g DW), along with superior functional properties, including higher water and oil absorption capacities (638.81% and 425.85%, respectively) and foaming capacity and stability (25.67% and 58.48%). The investigation of volatile compounds found that *A. abyssinica* had higher amounts of hotrienol (4.53%), yomogi alcohol (3.92%), caryophyllene (3.67%), and carvotanacetone (3.64%), which possess anti‐inflammatory, antimicrobial, and antioxidant properties. *Artemisia abyssinica* contributed over 30% of the recommended dietary intake (RDI) of amino acids. It displayed superior levels of sodium (31.46 mg/100 g DW) and calcium (238.07 mg/100 g DW). It also exhibited higher levels of organic acids, particularly malic acid, butyric acid, and succinic acid, compared to *A. arborescens*. Fatty acid analysis revealed palmitic and linoleic acids as primary components in both plants, with *A. abyssinica* having a higher palmitic acid content. *Artemisia abyssinica* also had higher vitamin C and thiamine levels. Although *A. arborescens* showed the highest total phenolic content (TPC), antioxidant activity, and capacity, *A. abyssinica* demonstrated acceptable efficiency in TPC and antioxidant content. These findings highlight the potential of both *Artemisia* species, particularly *A. abyssinica*, as valuable sources of nutrients and bioactive compounds for various applications.

## Graphical abstract

The study found that *Artemisia abyssinica* has superior nutritional and functional qualities than *Artemisia arborescens*, including more fat, fiber, carbohydrate content, and energy. *Artemisia abyssinica* also has high water and oil absorption capacity, amino acid content, and amounts of sodium, calcium, organic acids, and vitamins. Despite *A. arborescens*' higher total phenolic content and antioxidant activity, *A. abyssinica*'s significant bioactive components and nutritional profile indicate its potential as a functional nutritional supplement.

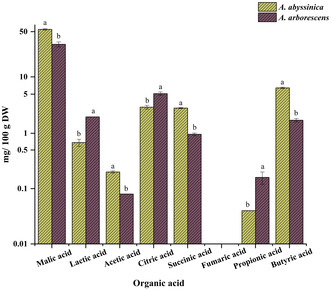

## 1 INTRODUCTION

Wild edible plants offer a wide range of essential macro‐ and micronutrients to enhance the diets of people (Bhatti et al., 2022; Ullah et al., 2023). Nutritional evaluations of wild edible plants frequently revealed nutrient contents comparable to or superior to cultivated varieties, rendering them crucial sources of nutrients, fatty acids, and vitamins in diets (Ullah & Badshah, 2023). Furthermore, the nutritional profiling of various wild edible plants has revealed high levels of proteins, fats, minerals, amino acids, and antioxidants, indicating their potential as future foods and sources for developing dietary supplements and nutraceuticals (Bhatti et al., 2022; Talang et al., 2023). Also, health‐promoting biological activities, phytochemical profiles, and nutritional composition are essential when evaluating the therapeutic applications of chosen medicinal plants (Kumar et al., 2021). So, the nutritional properties of plants not only enhance sustainability but also offer opportunities to widen the food chain of native populations, highlighting the importance of evaluating and utilizing wild plants for their nutritional benefits (Talang et al., 2023), and several studies suggest further investigation into the levels of bioactive compounds and nutritional factors within wild and medicinal plants for understanding their potential applications in the food and drug industries (A Aziz & Mhd Jalil, 2019; Arenas‐Salazar et al., 2024; Siddiqui et al., 2024).

The genus *Artemisia* is a large group of plants in the Asteraceae family, with about 500 species worldwide (Umam et al., 2023). Previous investigations have reported an extensive range of biological properties, including antioxidant, antimalarial, antibacterial, antihepatotoxic, antifungal, and cytotoxic, for the many *Artemisia* species (Mohammed et al., 2022). Some species of *Artemisia*, such as *Artemisia abyssinica*, are used locally to treat a variety of diseases, including leprosy, rabies, tonsillitis, gonorrhea, cough, and syphilis (Al‐Maqtari, Al‐Ansi, et al., 2021). For the treatment of domestic animal epilepsy, fresh roots of *A. abyssinica* are also used as juice (Tesfay et al., 2022). Also, tannins, saponins, flavonoids, alkaloids, essential oils (EOs), and phenolic compounds were the main phytochemicals identified in *A. abyssinica* (Achamo et al., 2022). *Artemisia arborescens* is another important medicinal species in the *Artemisia* group that has been used in human and veterinary medicine since ancient times for its antidiabetic, anti‐inflammatory, antipyretic, tonic, antispasmodic, sedative, and abortifacient properties (Russo et al., 2023). Recent studies have shown that EOs of *A. arborescens* protect against nephrotoxicity, herpesvirus infection, and inflammation (Riahi et al., 2019). Recently, *A. arborescens* was used to create silver nanoparticles that showed varying levels of growth inhibition on cancer cells, suggesting their potential as an anticancer treatment (Bordoni et al., 2021). On the other hand, the fatty acid content of *Artemisia* varies based on the species. A study on eight types of *Artemisia* plants revealed that the leaves of these plants contain high levels of fatty acids compared to some leafy vegetables, including linolenic acid (C18:3), linoleic acid (C18:2), oleic acid (C18:1), palmitoleic acid (C16:1), and palmitic acid (C16:0). The total lipid content in the leaves ranged from 3.31 to 17.78 mg of fatty acids per gram of fresh weight (Carvalho et al., 2011). Also, an analysis of phytochemicals in five different species of *Artemisia* found that unsaturated fatty acids, including those with antimalarial qualities like linoleic acid, arachidonic acid, and linolenic acid, made up 60% of the total, and saturated fatty acids formed about 40% (Kursat et al., 2015). Consequently, these plants may serve as crucial suppliers of essential fatty acids since humans are unable to synthesize them internally and must obtain them from their diet (Rizzo et al., 2023).

Although these plants have many unique characteristics, their plant contents for nutritional and therapeutic purposes and prospective use as supplements have not been sufficiently studied. So, the primary aim of this study was to examine the nutritional profiles, physical attributes, bioactive properties, and chemical compositions of these plants, including fatty acid, mineral, mono‐ and disaccharides, water‐soluble vitamin, amino acid, and organic acid analysis, to evaluate their potential applications as medicinal aids, nutritional supplements, and everyday dietary sources.

## 2 MATERIALS AND METHODS

### 2.1 Materials

Gallic acid was sourced from Shanghai Yuanye Bio‐Technology Co., Ltd. in China. The compound 2,2‐diphenyl‐1‐picrylhydrazyl (DPPH^•^) was obtained from J & K Scientific Co., Ltd. Trolox was supplied by Sigma‐Aldrich in the United States. Ascorbic acid (AA) was acquired from Sinopharm Chemical Reagent Co., Ltd. in China. The remaining chemicals were procured from Sinopharm Chemical Reagent Co., Ltd. in China. All media and chemicals were preserved under optimized conditions. Analytical grade chemicals were consistently employed throughout the study.

### 2.2 Sample collection and preparation

The aerial parts of *A. abyssinica* and *A. arborescens* were collected from the Al‐Ahjur region, Sana'a, Yemen. This region is located at around 15°.468796 N latitude and 43°.88165 E longitude, with an elevation of around 2300 m. The collected plant specimens were identified based on morphological characteristics and comparison with preserved herbarium samples in the Biology Department at Sana'a University. The aerial parts of the plants were washed with tap water to eliminate debris, followed by 10 days of drying at room temperature, away from sunlight, until complete dryness was achieved. Afterward, the dried parts were ground into powder using an electric mill and stored in sealed polyethylene bags at 4°C for future use (Al‐Maqtari, Al‐Ansi, et al., 2021).

### 2.3 Nutritional composition and energy calculation

The nutritional profiles of *A. abyssinica* and *A. arborescens* samples were assessed using the established methods in AOAC (2011). Parameters such as moisture content, protein (AOAC, 960.52), ash (AOAC, 940.26), and fat (AOAC, 920.85) were quantified. All results were presented as gram per 100 g of dry weight (g/100 g DW), except for energy, which was expressed as kilocalories/100 g DW (kcal/100 g DW).

#### 2.3.1 Crude protein content

The protein content of a 2 g sample was analyzed using an Automatic Kjeldahl Analyzer (SH220N Graphite Digester, K9840 Auto Kjeldahl Distillation Unit; Hanon Instruments, China) using the micro‐Kjeldahl procedure (AOAC, 960.52). The protein content was calculated by converting the nitrogen content to a percentage using a N × 6.25 conversion factor.

#### 2.3.2 Fiber content

The crude fiber content was determined using the methodology outlined by Mohammed et al. (2019). Briefly, a solution of 1.25% H_2_SO_4_ (v/v) with a volume of 200 mL was heated to its boiling point. This solution was then employed to digest a 2 g sample for 30 min. Subsequently, the mixture was heated for 30 min in a 200‐mL solution containing 1.25% NaOH (w/v). The resulting mixture was then filtered, dried, and subjected to ashing. Equation (1) was utilized to calculate crude fiber.

(1) $$\text{Crude fiber}\;\left(\%\right)=\frac{\text{Weight of residues}-\text{Weight of}\;\mathrm{ash}}{\text{Weight of sample}}$$

#### 2.3.3 Crude fat content

The crude fat content of a dried sample weighing 3 g was evaluated by extracting it with petroleum ether using the Soxhlet extraction technique (AOAC, 920.85) with the Automatic Soxhlet Extractor (SOX606 Auto Fat Analyzer; Hanon Instruments, Jinan, China). The crude fat content was determined by calculating the amount of fat in the sample relative to its DW.

#### 2.3.4 Ash content

The ash content was determined using the AOAC (940.26) standard technique. A muffle furnace (JNL‐12XB; Luoyang Liyu Furnace Co., Ltd., Luoyang, China) was employed to burn 1 g of plant powder until it reached a consistent weight at a temperature of 525°C. The experiment was replicated three times, and the ash content was determined using DW.

#### 2.3.5 Total carbohydrate content

The carbohydrate content, based on DW, was determined by subtracting the total percentages of protein, fat, and ash from 100% using Equation (2).

(2) $$\text{Total carbohydrate}=100-\left(m_{P}+m_{L}+m_{W}+m_{A}\right)$$

where *m*_P_ = content of crude protein; *m*_L_ = content of crude lipid; *m*_W_ = moisture content; *m*_A_ = content of ash.

#### 2.3.6 Calculation of energy

The protein, fat, and carbohydrate statistics were utilized to calculate energy levels using average factors of 4, 9, and 4 kcal/g, respectively.

### 2.4 Physical properties

#### 2.4.1 Solubility

The water solubility of the plant sample was evaluated following the procedure outlined by Al‐Bukhaiti et al. (2019), with slight adjustments. Initially, 1 g of powdered plant sample was dispersed in 50 mL of deionized water within a centrifuge tube. The mixture was subjected to magnetic stirring at 500 rpm for 30 min at room temperature. Subsequently, centrifugation was performed at 5000 rpm for 5 min to separate the supernatant. The collected supernatant was then transferred to a crucible and subjected to overnight drying in a 105°C oven. The solubility percentage was determined using Equation (3):

(3) $$\text{Solubility}\;\left(\%\right)=\frac{\text{Weight of dried supernatant}\;\left(g\right)}{\text{Weight of the original powder}\;\left(g\right)}\times 100$$

#### 2.4.2 Water activity (*a*_w_)

The water activity of the samples was measured using a Labmaster‐aw water activity meter from Switzerland. First, a gram of plant sample powder was uniformly spread inside a plastic cup provided with the device. Subsequently, the cup containing the sample was placed in the designated chamber of the water activity meter. After allowing sufficient equilibration time, the water activity measurement was conducted 10–15 min after placing the sample in the device, as recommended in previous studies (Al‐Maqtari, Mohammed, et al., 2021).

#### 2.4.3 Hygroscopicity behavior

The hygroscopicity of the plant powders was assessed following the method outlined by Mahdi, Mohammed, et al. (2020). Initially, a gram of each plant sample powder was placed in a sealed container at a controlled temperature of 25°C alongside a saturated sodium chloride solution to maintain a consistent relative humidity of 75.28%. After a week‐long incubation period, the samples were carefully removed from the containers and weighed to determine any changes in mass due to hygroscopic absorption. The hygroscopic behavior of the samples was quantitatively assessed using Equation (4):

(4) $$\text{Hygroscopicity}\;\left(\%\right)=\frac{\text{Adsorbed moisture}\;\left(g\right)}{\text{Sample weight}\;\left(g\right)}\times 100$$

#### 2.4.4 Bulk, tapped, and particle density

The measurement of bulk (*ρ*_b_), tapped (*ρ*_t_), and particle (*ρ*_p_) density of plant samples followed the methodology outlined by Mohammed et al. (2021). First, *ρ*_b_ was determined by introducing 2 g of the samples into a 25‐mL measuring glass cylinder. To ensure accuracy and eliminate adhesion powder on the cylinder walls, gentle tapping was applied. The *ρ*_b_ values were computed based on the mass (g) ratio to volume (cm^3^).

The *ρ*_t_ was determined using the same measuring glass cylinder. The cylinder containing the sample was tapped manually approximately 100 times. This tapping involved lifting the cylinder and allowing it to descend onto a flat surface from a standard height of 10 cm. After tapping, the powder volume was directly read from the cylinder. The *ρ*_t_ was calculated by dividing the mass of the sample (g) by the volume (cm^3^) it occupied post‐tapping.

*ρ*_p_ was established by weighing 1 g of plant samples and placing them within a sealed graduated glass tube (10 mL capacity). Subsequently, 6 mL of petroleum ether was added into the tube, and agitation was applied to ensure the dispersion of any air bubbles. An additional 1 mL of petroleum ether was added to the tube to remove any particles adhering to the inner wall. The calculation of *ρ*_p_ values was subsequently conducted using Equation (5):

(5) $$\;\rho_{p}=\frac{\text{Powder weight}\;\left(g\right)}{\text{The total volume of petroleum ether with suspended powder}\;\left({\mathrm{cm}}^{3}\right)-7}$$

#### 2.4.5 Cohesiveness and flowability

The cohesion and flow properties of the samples were assessed following established methods outlined by Düsenberg et al. (2023). Cohesion was evaluated using the Hausner ratio (HR), and the flowability of the samples was determined using Carr's index (CI). The calculation of HR and CI involved utilizing the *ρ*_b_ and tapped density *ρ*_t_ values, as described by Equations (6) and (7), respectively.

(6) $$\mathrm{HR}=\frac{\rho_{t}}{\rho_{b}}$$

(7) $$\mathrm{CI}\;\left(\%\right)=\frac{\rho_{t}-\rho_{b}}{\rho_{t}}\times 100$$

#### 2.4.6 Porosity

The plant sample porosity was determined according to the method described by Mahdi et al. (2021), utilizing Equation (8) with the provided values of *ρ*_t_ and *ρ*_p_.

(8) $$\text{Porosity}\;\left(\%\right)=\frac{\rho_{p}-\rho_{t}\;}{\rho_{p}}\times 100$$

#### 2.4.7 pH measurement

The pH measurement was achieved using a pH meter, following the methodology conducted by Al‐Bukhaiti et al. (2019). Initially, a gram of the powdered plant sample was combined with 100 mL of deionized water and vortexed for 3 min to ensure thorough mixing. Subsequently, the mixture was allowed to stand at ambient temperature for 1 h to facilitate equilibration. After incubation, the mixture was centrifuged at 4000 rpm for 20 min to separate the supernatant. The pH of the resulting supernatant was then measured.

#### 2.4.8 Color measurement

The lightness (L*), redness (a*), and yellowness (b*) attributes of plant sample powder were determined following the method outlined by Hawa et al. (2021), utilizing a Hunter‐Lab Ultra‐Scan PRO spectrophotometer from Hunter Associates Laboratory, Inc., Virginia, USA. The total color variation (ΔE*) was then computed using Equation (9):

(9) $$\Delta E^{*}={\left[{\left(L^{*}-L^{0}\right)}^{2}+{\left(a^{*}-a^{0}\right)}^{2}+{\left(b^{*}-b^{0}\right)}^{2}\right]}^{1/2}$$

where L* − L ^0^, a* − a ^0^, and b* − b ^0^ represent the differences in color parameters between the sample (*L* ^0^, *a* ^0^, and *b* ^0^) and the standard (L* = 92.94, a* = −0.9, and b* = 0.63).

### 2.5 Functional properties

#### 2.5.1 Water absorption capacity and oil absorption capacity

The water absorption capacity (WAC) and oil absorption capacity (OAC) were determined using a modified version of the methodology summarized by Sangokunle et al. (2020). First, measured quantities of 1 g of each sample were placed into preweighed centrifuge tubes. For the WAC test, 10 g of deionized water was added to each tube, while 10 g of refined sunflower oil was added for the OAC test. The samples were mixed with vortex for 10 s at 5‐min intervals over 30 min, maintaining a constant temperature of 25°C. Following the mixing period, the samples were centrifuged at 4000 rpm for 25 min to facilitate the separation of the unabsorbed liquid from the solid matrix. Subsequently, the supernatant was carefully decanted, and the remaining sample was reweighed to determine the extent of liquid absorption. The difference in weight between the initial dry sample and the final wet sample was then calculated to quantify the water and oil absorption capacities.

#### 2.5.2 Foaming capacity and foaming stability

The foaming capacity (FC) and foaming stability (FS) of plant samples were measured using the approach described by Khalid and Elhardallou (2015), with some modifications. Initially, 2 g of powdered plant samples were weighed and transferred into a 500‐mL beaker. Subsequently, 100 mL of deionized water was added to the plant sample. The contents were then thoroughly mixed using a digital homogenizer (Model T25DS25; IKA Industrie, Königswinter, Germany) at 15,000 rpm for 6 min at a controlled temperature of 25°C to ensure uniform dispersion of the sample in the water. After completion of the homogenization process, the volume of foam generated was promptly measured by transferring the contents to a 250‐mL measuring cylinder, enabling the determination of FC. Following this, the stability of the foam was assessed by monitoring the reduction in foaming ability after 1 h.

### 2.6 Volatile composition analysis via GC–MS

Volatile compounds were analyzed in line with the methodology outlined by Al‐Maqtari, Al‐Ansi, et al. (2021) using a gas chromatography–mass spectrometry (GC–MS) setup, specifically the Scion SQ 456‐GC instrument from Bruker, USA. A DB‐Wax column measuring 30 m in length and 0.25 mm in diameter, with a 0.25‐μm film (J&W Scientific, Folsom, CA, USA), was employed for separation. Helium gas was utilized as the mobile phase at a flow rate of 0.80 mL/min. Volatile compounds were identified by matching their mass spectra to established compounds listed in mass spectrometry libraries, namely Wiley (New York, USA) and the National Institute of Standards and Technology in Gaithersburg, Maryland, USA. The classification of volatile compounds followed the guidelines of the Volatile Compounds in Food online database (16.4 version, February 13, 2017).

### 2.7 Amino acid analysis procedure

The total amino acid determination was conducted using the method outlined by Amadou et al. (2013) with slight modifications. The sample (1 g) was digested in 10 mL of 6 M HCl at 110°C under a nitrogen atmosphere for 22 h. After digestion, the solution was dissolved in 7.5 mL of sodium citrate buffer (pH 2.2), followed by filtration and centrifugation at 4000 rpm for 15 min. A 10‐μL aliquot of the resulting solution was injected into a reverse‐phase high‐performance liquid chromatography (RP‐HPLC) Agilent 1100 (Palo Alto, CA, USA) system. This system was equipped with an Agilent Hypersil ODS column (250 mm length, 4.0 mm diameter, 5 μm particle size) and a UV detector with variable wavelength capabilities. UV detection was set at 338 nm and 262 nm for detecting proline. The chromatographic separation was achieved using two mobile phases: mobile phase A, consisting of 27.6 mmol/L tetrahydrofuran/triethylamine/sodium acetate (2.5:0.11:500, v/v/v) at pH 7.2, and mobile phase B, composed of 80.9 mmol/L acetonitrile/methanol/sodium acetate (2:2:1, v/v/v) at pH 7.2. The mobile phase flowed at a rate of 1 mL/min. Amino acid types and concentrations are expressed in mg/100 g DW.

### 2.8 Mineral element analysis

The mineral element concentrations were assessed following the methodology outlined by Al‐Bukhaiti et al. (2019), with minor adjustments. Initially, pre‐prepared ash samples were digested using a mixture of HNO_3_/HCl/H_2_O (1:2:3, v/v/v). The resulting digested sample solution was then filtered, and deionized water was added to achieve a final volume of 25 mL. Atomic absorption spectroscopy (Varian spectra AA‐220 FS, Australia) was employed to analyze the elements, including cobalt (Co), nickel (Ni), manganese (Mn), copper (Cu), iron (Fe), zinc (Zn), potassium (K), calcium (Ca), sodium (Na), and magnesium (Mg). The flame mode was utilized, with absorption signals measured during the atomization step based on peak height. An air–acetylene flame was utilized for sodium and potassium determination, while a nitrous oxide–acetylene flame was used for the other elements. The spectral wavelengths of the instrument were 422.7 nm for Ca, 240.7 nm for Co, 324.8 nm for Cu, 248.3 nm for Fe, 766.5 nm for K, 285.2 nm for Mg, 279.5 nm for Mn, 589 nm for Na, 232 nm for Ni, and 232 nm for Zn. The instrument provided three slit widths with spectral band passes of 0.1, 0.2, and 0.5 nm, and lamp currents ranged from 1 to 6 mA. After serial dilutions, calibration curves were prepared using analytical grade stock standard solutions (1000 ppm). The mineral content was quantified as mg/100 g DW.

### 2.9 Quantification of organic acid composition

The organic acid content was quantified using the approach described by Ghamry et al. (2022). The concentration of organic acids was established through RP‐HPLC. First, 0.2 g of plant sample powder was mixed with 10 mL of distilled water for 3 min. The resulting mixture was then vortexed and filtered using a 0.45‐μm microfilter. This filtered solution (5 μL) was introduced into an RP‐HPLC system (Agilent 1100, Palo Alto, CA, USA) featuring a Diamonsil C‐18 column (4.6 mm × 250 mm, 5 μm) and UV detection set at 210 nm. The mobile phase, a solution of H_3_PO_4_/H_2_O/CH_3_OH (0.05:95:5, v/v/v), was flowed at 0.8 mL/min, and organic acid quantities were expressed as mg/100 g DW.

### 2.10 Fatty acid composition determination

The quantitative analysis of fatty acid content was conducted utilizing the methodology outlined by Albakry et al. (2022). The fatty acid composition of the plant sample powder was analyzed using GC. Each sample (20 mg) was mixed with 2 mL of hexane and 500 μL of a 2 M KOH‐CH_3_OH solution in a sealable tube. The mixture was vortexed for 2 min, followed by adding 5 mL of saturated NaCl solution. The upper layer was then collected, dehydrated with anhydrous sodium sulfate, and centrifuged for 3 min at 10,000 rpm. Subsequently, 100 μL of the resulting solution containing fatty acid methyl esters was analyzed using a GC (model 7820A; Agilent Technologies USA) equipped with a hydrogen flame ionization detector.

### 2.11 Measurement of water‐soluble vitamins

The concentration of water‐soluble vitamins was assessed following the method outlined by Heudi et al. (2005). Initially, 2 g of the sample were accurately weighed and placed into a 100‐mL volumetric amber flask. Subsequently, 40 mL of water and 4 mL of 2 M NaOH were added to the flask, and the suspension was vigorously shaken. The pH of the resulting solution was then adjusted to 7 by adding 50 mL of 1 M phosphate buffer (pH 5.5). The flask was filled to the mark with distilled water and subjected to 10 min of sonication in an ultrasonic bath to ensure thorough mixing and dissolution. Following sonication, the diluted solution was filtered through a 0.45‐μm filter to remove any particulate matter. Subsequently, 10 μL of the filtrate was directly injected into the RP‐HPLC (Agilent 1100, USA) equipped with a Symmetry C18 Column (250 mm length, 4.6 mm diameter, 5 μm particle size). The system was operated at 30°C with a linked UV detector set at 280 and 254 nm wavelengths to evaluate the water‐soluble vitamin content. The RP‐HPLC system employed a mobile phase consisting of two components: mobile phase A (CH_3_OH/H_2_O/H_3_PO_4_ with a mixing ratio of 5:95:0.05, v/v/v) and mobile phase B (CH_3_OH/H_2_O/H_3_PO_4_ with a mixing ratio of 80:20:0.05, v/v/v). The system utilized a gradient elution program with a flow rate of 0.8 mL/min and time intervals of 0, 20, and 25 min. To verify the accuracy of the results, vitamin standards were prepared in the mobile phase, and peaks were authenticated by comparing them with standard vitamin peaks. The quantification of vitamins was performed by calculating the peak areas relative to those of the standard vitamin peaks. The results of the water‐soluble vitamins were expressed as mg/100 g DW of the sample.

### 2.12 Quantification of mono‐ and disaccharides

The sugar composition of tested plant samples was determined following the method outlined by Mahdi, Al‐Ansi, et al. (2020), with slight modifications. In brief, 1 g of each sample was weighed and treated with 10 mL of an 80% (v/v) aqueous ethanol solution. The mixture was agitated at 80°C for 30 min using a mechanical shaker. After centrifugation at 4000 rpm for 10 min, the resulting supernatant was filtered through a 0.45‐μm filter. Then, it was injected (10 μm) into a high‐performance liquid chromatography (HPLC) system (Waters Corporation, model 1525, MA, USA). The HPLC system was equipped with a binary HPLC pump (Waters, Sugarpak I Column [6.5 mm × 300 mm]) and a refractive index (RI) detector set at 85°C. The mobile phase employed was pure water, flowing at a 0.3‐mL/min rate. The peaks were confirmed by incorporating standard sugars into specific samples, and the area of each peak was determined according to the area of the standard sugar peaks. The obtained saccharide data were presented as mg/100 g DW.

### 2.13 Bioactive compounds

#### 2.13.1 Extraction method

Bioactive properties were evaluated using an ethanolic extract derived from plant powder. Briefly, the plant sample powder was combined with absolute ethanol at a 1:20 (w/v) ratio through shaking. Following agitation, the mixture underwent centrifugation at 10,000 rpm for 10 min before filtration. The resulting supernatant was utilized for assessing total phenolic content (TPC), antioxidant activity using DPPH^•^, and antioxidant capacity using 2,2′‐azino‐*bis*(3‐ethylbenzothiazoline‐6‐sulfonic acid) ABTS^•+^‐SE as described by Al‐Maqtari, Al‐Ansi, et al. (2021), with minor adjustments.

#### 2.13.2 Determination of TPC

The TPC of the plant extract was assessed using a 24‐well microplate assay based on the Folin–Ciocalteu colorimetric method. Concisely, 100 μL of the plant extract diluted to a concentration of 5 mg/mL and 1.65 mL of Folin phenol reagent with a concentration of 0.2 N were added to each well of a 24‐well microplate. After 5 min, a volume of 1.35 mL of a sodium carbonate solution with a concentration of 7.5% was introduced into the combination. The mixture was left undisturbed at room temperature for 105 min without light. Deionized water served as a reference sample with no measurable properties. The microplate reader assessed the absorbance at 765 nm after incubation. The results were quantified as mg of gallic acid equivalent per g of DW (mg GAE/g DW) using the linear equation *Y* = 0.0057*x* + 0.0644, with an *R* ^2^ value of 0.9976.

#### 2.13.3 Antioxidant activity assessment through DPPH ^•^‐SA assay

The DPPH^•^ method was employed to assess the ability of plant extracts to scavenge free radicals. In summary, a 24‐well microplate was utilized, and 25 μL of a diluted plant extract (5 mg/mL) was added to wells containing 1.75 mL of a methanolic DPPH^•^ solution (60 μM). This mixture was then thoroughly mixed and kept in the dark for 30 min at room temperature. Following incubation, the absorbance of samples was measured at 517 nm, and outcomes were presented as mg AA equivalents per g of DW (mg AA/g DW). The quantification was accomplished through the linear AA equation (*y* = −0.0692*x* + 0.5932 with *R* ^2^ = 0.991).

#### 2.13.4 Measurement of antioxidant capacity using the ABTS ^•+^‐SE assay

The ABTS^•+^‐SE assay was employed to assess the antioxidant capacity of plant extracts utilizing a 24‐well microplate. The ABTS stock solution (7 mM) was prepared by dissolving 38.41 mg of ABTS in 10 mL of deionized water and subsequently mixing it at a 1:1 (v/v) ratio with the potassium persulfate solution (2.45 mmol) to create the ABTS^•+^ solution. The solution was incubated for 16 h at room temperature without exposure to light until it formed a stable blue‐green cation radical solution. It was then diluted with ethanol until it attained an absorbance of 0.7 ± 0.2 at 734 nm before use. Subsequently, the ABTS^•+^ ethanolic solution (1.75 mL) was introduced into each well of a 24‐well microplate, along with 100 μL of the diluted plant extract (5 mg/mL). The mixture was homogenized and stored in a lightless environment at room temperature for 10 min. The sample was replaced with deionized water as a blank. Following incubation, the absorbance was measured at 734 nm using a microplate reader. The antioxidant capacity was calculated using the linear equation of Trolox ([*y* = −0.0274*x* + 0.456 with *R* ^2^ = 0.9974]), yielding results in mg Trolox equivalents per g of DW (mg TE/g DW).

### 2.14 Statistical analysis

Data analysis employed Stat‐Packets Statistical Analysis software (SPSS Base 20.0; SPSS, Inc., Chicago, IL). Triplicate testing was conducted for each sample. Independent sample *t* tests assessed result disparities, with significance attributed to mean differences at *p* < .05.

## 3 RESULTS AND DISCUSSION

### 3.1 Nutritional composition and energy calculation

The nutritional composition, energy values, and moisture content of *A. abyssinica* and *A. arborescens* are presented in Table 1. The analysis revealed significant differences (*p* < .05) between the two plant types regarding crude fat, fiber, ash, total carbohydrate, and energy content. However, no significant difference was observed in their crude protein and moisture content. *Artemisia abyssinica* exhibited higher levels of crude fat (4.76 ± 0.04 g/100 g DW), fiber (16.07 ± 0.91 g/100 g DW), and carbohydrate (55.87 ± 0.48 g/100 g DW) compared to *A. arborescens*, with increments of 104.29%, 25.253%, and 52.06%, respectively. This suggests that *A. abyssinica* could be a more favorable choice for people who desire a higher consumption of fat and dietary fiber, as well as a potentially higher carbohydrate content per serving. Conversely, *A. arborescens* displayed a greater ash content (20.03 ± 0.01 g/100 g DW) than *A. abyssinica*, showing a 41.86% increase. In the same context, *A. abyssinica* exhibited a notably greater energy content (302.15 kcal/100 g DW) compared to *A. arborescens* (279.09 kcal/100 g DW), which aligns with the disparities in fat and carbohydrate composition. This observation suggests that *A. abyssinica* may offer a more potential alternative as a power source. Other nutritional parameters showed negligible variation between the two plant species. Nevertheless, the crude protein, crude fat, carbohydrate, and fiber findings aligned with previous reports on the nutritional composition of *Artemisia maritima* (13.17%, 4.11%, 49.63%, and 21.06%, respectively) (Zaman et al., 2022), as well as comparable trends observed in wild food plants such as *Cotoneaster nummularia* (12.4%, 17.00%, 65.33%, and 15.50%), *Ziziphora clinopodioides* (12.7%, 2.16%, 46.00%, and 19.33%), and *Rumex hastatus* (9.7%, 3.50%, 42.05%, and 13.90%) (Ullah & Badshah, 2023). In the same context, the ash content was closer to that reported for wild edible plants such as *Theileria orientalis* (15.89%–18.73%) and *Polygonum cognatum* (13.09%–15.71%) (Kibar & Kibar, 2017), but higher than those values reported for related plants such as *Artemisia annua* (3.43%–10.60%) (Brisibe et al., 2009), *Artemisia absinthium* (8.64%–9.11%) (Yousefi et al., 2021), *A. maritima* (6.32%) (Zaman et al., 2022), and lower than the values reported by Mayuri et al. (2022) for *Artemisia stelleriana* (29.74%). Plant proteins offer various health benefits, including aiding in cell repair, immune responses, and muscle maintenance. The consumption of plant‐based proteins has been shown to contribute to the reduction of various health conditions such as obesity, high blood pressure, type 2 diabetes, high cholesterol, and specific types of cancer, including breast, ovarian, and colorectal cancers (Yıkmış et al., 2019). From a nutritional perspective, the optimal incorporation of proteins derived from these plants can provide a sufficient quantity of vital amino acids needed for the requirements of human health, offering a diverse range of applications, including food supplements, edible coatings, emulsifiers, and sources of bioactive peptides (Kumar et al., 2022). On the other hand, plant fibers contribute to gastrointestinal health by assisting in the removal of toxic waste products (Snauwaert et al., 2023). Certain fatty acids derived from plant fats, such as monounsaturated and polyunsaturated fats, have been associated with cardiovascular benefits, including reducing the risk of heart disease (Sherratt et al., 2023). Also, the secondary plant metabolites, including polyphenolic chemicals and carotenoids, have antioxidant and bioactive effects. So, the secondary metabolites of these plants may lower blood pressure, Alzheimer's disease protein levels, urinary tract infections, and cancer risk (Aluko, 2011).

**TABLE 1:** Nutritional analyses (g/100 g DW), energy (kcal/100 g DW), moisture content (%), pH, mono‐ and disaccharides of *Artemisia abyssinica* and *Artemisia arborescens* plant powders.

	*A. abyssinica*	*A. arborescens*
Crude protein	14.91 ± 0.17^a^	15.39 ± 0.24^a^
Crude fat	4.76 ± 0.04^a^	2.33 ± 0.03^b^
Fiber	16.07 ± 0.91^a^	12.83 ± 0.09^b^
Ash	14.12 ± 0.41^b^	20.03 ± 0.01^a^
Total carbohydrate	55.87 ± 0.48^a^	52.06 ± 0.33^b^
Energy	302.15 ± 1.67^a^	279.09 ± 0.28^b^
Moisture content	10.34 ± 0.01^a^	10.20 ± 0.06^b^
pH	5.67 ± 0.00^a^	5.64 ± 0.00^b^
Sucrose	146.72 ± 8.53^a^	135.1 ± 3.11^b^
Glucose	58.07 ± 2.41^a^	42.5 ± 1.08^b^
Fructose	602.13 ± 32.12^a^	NF

*Note*: Different letters show significant changes (*p* < .05) in the same line; values are the means ± standard deviation of three determinations.

Abbreviation: NF, not found.

However, the nutrient content within plants is influenced by several factors, such as soil type, moisture levels, and the developmental stage of the crop (Tsehay et al., 2023). Soil physical conditions such as water content, temperature, aeration, and root development play a crucial role in the absorption of nutrients by plant roots (Reichardt et al., 2020). Additionally, the mobility of nutrients within plants varies, with nitrogen being highly mobile compared to phosphorus, potassium, and magnesium, affecting the distribution of nutrients within the plant body (Singh & Tiwari, 2018). Furthermore, essential mineral elements, both macronutrients and micronutrients, are vital for plant growth, development, and stress tolerance, with deficiencies or excess concentrations impacting plant health and growth (Shrivastav et al., 2020). Organic matter and organic–mineral manure can also influence nutrient availability by supporting microbial populations that aid in nutrient solubilization and mineralization (García et al., 2007).

The outcomes in Table 1 also indicated that the moisture content of the *A. abyssinica* sample (10.34%) and *A. arborescens* sample (10.20%) aligned with the moisture content of four wild edible fruits, which ranged from 9.83% to 13.10% (Jiru et al., 2023), while higher than reported for *A. maritima* (5.72%) (Zaman et al., 2022) and lower than *A. stelleriana* (21.95%) (Mayuri et al., 2022). These findings demonstrate that the moisture content of tested plants was below the recommended range (12%–13%), which ensures optimal storage stability (Chukwu & Abdullahi, 2015). Excessive moisture in powders can foster microbial contamination, whereas lower moisture content supports prolonged storage and suitability for food and pharmaceutical processing applications (Otegbayo et al., 2018). Soil qualities such as bulk densities, water content, and pH values influence moisture characteristics (Wo et al., 2010). Additionally, the soil water content and its availability to plants are crucial, with field capacity and saturation water content being affected by soil texture and structure (Orfánus & Eitzinger, 2010). Furthermore, the moisture content is influenced by internal moisture, specific surface area, and ultrafine particles affecting water drainage (Edeh & Mašek, 2022). Moreover, plant type, grassland type, and seasonality are key factors (Tiemeyer et al., 2024). Arbuscular mycorrhizal fungi also help plants manage water, and the arbuscular mycorrhizal symbiosis is often seen as a way for host plants to recover from dry stress (Püschel et al., 2020).

### 3.2 Physical properties

#### 3.2.1 Solubility, water activity, and hygroscopic behavior

**FIGURE 1:**
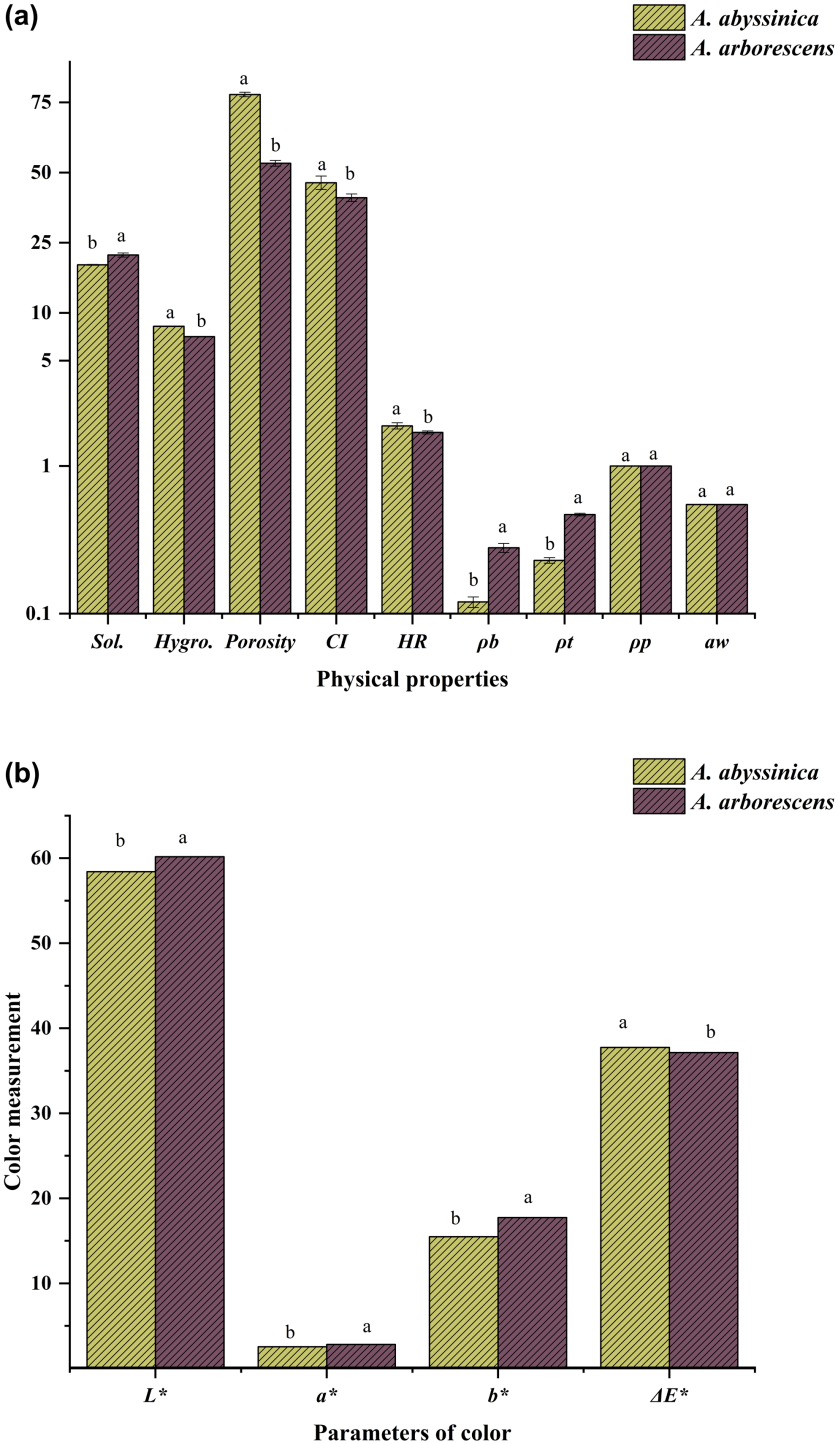
(A) Physical properties and (B) color values of *Artemisia abyssinica* and *Artemisia arborescens* plant powders. CI, Carr's index; HR, Hausner ratio; Hygro., hygroscopicity; Slo., solubility; *ρ*_b_, bulk density; *ρ*_p_, particle density; *ρ*_t_, tapped density; L* = lightness; a* = redness; b* = yellowness; ΔE* = the total color variation. The different letters indicate that the values differ substantially (*p* < .05).

The solubility, water activity, and hygroscopic behavior of the tested plant samples are depicted in Figure 1A. The results revealed that *A. arborescens* had the highest solubility at 21.57 ± 0.57%, surpassing *A. abyssinica* with a solubility of 19.08 ± 0.12%. Conversely, *A. abyssinica* displayed a greater hygroscopicity value at 8.26 ± 0.04% compared to *A. arborescens* at 7.11 ± 0.01%. Comparable findings were reported by Al‐Bukhaiti et al. (2019), who reported a solubility of 18.74% for *Cissus rotundifolia* leaf powder. They suggested that the milling procedure causes the disintegration of starch granules, thereby enhancing solubility. Additionally, the outcomes of this investigation mirrored those documented by Embaby and Rayan (2016), who observed a solubility of 20.6% for acacia seed flour. They claimed that the composition of starch, proteins, and carbohydrates present in the sample could influence the water solubility index.

However, the solubility characteristics of different plant types vary based on their compositions. *Brassica juncea*, *Brassica napus*, and *Sinapis alba* seeds showed differences in solubility due to factors like pH and salt presence (Wanasundara et al., 2012). *Viscum album* L. triterpene acids exhibited varying solubilities in water and alkaline solutions (Jäger et al., 2007). Natural phenolic compounds displayed different solubilities in plants at various temperatures, influenced by factors like hydrogen bonding and crystal structures (Queimada et al., 2009). Also, biomolecules such as amino acids and sugars have diverse solubilities in water and mixed solvents, affected by interactions like hydrogen bonding and charged species (Macedo, 2005). In contrast, the reduced solubility of *A. abyssinica* might be attributed to its elevated fiber content (Mohammed et al., 2019). These outcomes also align with those detailed in Table 1.

On the other hand, the lower hygroscopicity of *A. arborescens* could be linked to its larger particle size. Rezende et al. (2018) emphasized that larger particles tend to have lower hygroscopicity due to their reduced surface area exposed to water, causing decreased water absorption. Additionally, variations in porous surface structures contribute to differing water absorption rates based on wall structure and configurations (Al‐Maqtari, Mohammed, et al., 2021).

In the same context, Figure 1A demonstrated that *A. abyssinica* and *A. arborescens* exhibited identical water activity values (0.55). Nevertheless, the water activity level of these plants was below 0.6. According to Al‐Maqtari et al. (2022), this threshold indicates a state where the growth of microorganisms is prevented. Tapia et al. (2007) reported that the growth of the majority of microorganisms can be inhibited when the water activity level is below 0.85. Therefore, storing these powders for a long period of time will be both safe and ideal. Similar findings were attained by Rafiq et al. (2023), who noted that elevating the air temperature during the drying process notably reduced the moisture content and water activity of the resulting powders. However, the fluctuations in water activity may be attributed to genotypes, agricultural factors, differences in cultivars, environmental conditions, and chemical compositions. Moreover, the sugar levels in the plants may decrease water activity (Pandiselvam et al., 2023). In addition, the moisture content affects the water activity, which affects the arrangement and interaction of powder particles, affecting the flowability (Juarez‐Enriquez et al., 2019).

#### 3.2.2 Bulk, tapped, and particle density

The *ρ*_b_, *ρ*_t_, and *ρ*_p_ values of the plants under study are presented in Figure 1A. Notably, *A. arborescens* exhibited the highest *ρ*_b_ and *ρ*_t_ values (0.28 ± 0.00 and 0.47 ± 0.01 g/cm^3^, respectively), surpassing those of *A. abyssinica* (0.12 ± 0.01 and 0.23 ± 0.01 g/cm^3^, respectively), with statistically significant differences (*p* < .05). However, no difference was observed in *ρ*_p_ values, which were 1.0 ± 0.00 g/cm^3^ for both plants. The results for *ρ*_b_ were similar to those reported by Coşkuner and Karababa (2007) for coriander (0.23–0.22 g/cm^3^) and lower than those of some wild edible plants reported by Kibar and Kibar (2017), who attributed these changes to variations in species and regional areas. However, powders with high *ρ*_b_ are more cost‐effective to ship and package, while powders with low *ρ*_b_, as affected by agglomeration, are another critical feature of instant powders (Sharma et al., 2012). So, the low value of *ρ*_b_ makes *A. abyssinica* appropriate for high‐nutrient density formulation of foods compared to *A. arborescens* (Al‐Bukhaiti et al., 2019), while the high *ρ*_t_ value makes *A. arborescens* appropriate for high packaging, transportation, and commercialization compared to *A. abyssinica*. Also, the *ρ*_t_ value of powders reflects characters like flowability, oxidation stability, and storage potential (Al‐Maqtari, Mohammed, et al., 2021). Conversely, *ρ*_p_ assumes significance as it aids in assessing weight and volume when selecting container sizes for high‐density versus low‐density dry products (Shahidi Noghabi & Molaveisi, 2020).

#### 3.2.3 Cohesiveness, flowability, and porosity

Figure 1A displays the HR, CI, and porosity of *A. abyssinica* and *A. arborescens* plant powders. The results showed that *A. abyssinica* had a higher HR (1.86 ± 0.09), CI (46.02 ± 2.62%), and porosity (77.24 ± 0.61%) than *A. arborescens* (1.68 ± 0.04, 40.28 ± 1.39%, and 53.63 ± 1.13%, respectively), with significant differences (*p* < .05). A lower HR indicates a more free‐flowing powder, while a higher CI indicates a more cohesive powder. In general, powders with high bulk porosity often exhibit poor flow behavior due to the larger interparticle distances (Mohammed et al., 2019). These statements are consistent with principles commonly used in the pharmaceutical and powder‐handling industries to assess the flow properties of powders and granular materials. These results suggest that *A. abyssinica* may be easier to handle and process than *A. arborescens*, while *A. arborescens* may be more suitable for applications where free‐flowing powders are required, such as in the pharmaceutical and food industries. Düsenberg et al. (2023) reported that low HR and CI values, indicative of high flowability, are associated with minimal differences between the *ρ*_b_ and *ρ*_t_ in powder materials.

On the other hand, the higher porosity of *A. abyssinica* suggests that it had a more open structure than *A. arborescens*. So, the higher porosity *of A. abyssinica* could have implications for its mechanical properties, such as its strength and stiffness. This could be due to several factors, such as the different particle size, shape, and surface roughness or the different ways in which the particles are arranged. For example, powders with small particles tend to have lower porosities than powders with large particles (Marczyk & Hebda, 2023). This is because small particles can pack more tightly together. Düsenberg et al. (2023) discovered similar results, stating that the density, apparent flowability, and handling of the powders must be classified as very cohesive according to particle sizes, inhomogeneity, and moisture content. However, the porosity of powders provides various advantages, such as improved particle strength, enhanced transfer mechanisms, greater absorption capacity, and increased specific surface area (Bram et al., 2022). As a result, it is well suited for a range of applications, such as controlled release systems, filtration, and adsorption. Conversely, it has a significant impact on various properties, including magnetic features, electrical and thermal conductivities, ultimate strength, elastic moduli, and other qualities that are closely associated with this feature (Ternero et al., 2021). So, it significantly impacts the heat and mass transfer processes in food materials and affects the nutritional, sensory, mechanical, and chemical characteristics of food (Joardder et al., 2016).

#### 3.2.4 Color and pH measurements

The results of the color measurements (L*, a*, b*) are given in Figure 1B. Significant differences (*p* < .05) were observed among the color values of the evaluated plants. The L*, a*, and b* values of the *A. arborescens* plant displayed 60.18 ± 0.01, 2.82 ± 0.02, and 17.73 ± 0.02, respectively, and were superior to those of *A. abyssinica* (58.41 ± 0.03, 2.54 ± 0.02, and 15.48 ± 0.01, respectively). The L* value is the degree of lightness, which ranges from 0 for black to 100 for white (Kibar & Kibar, 2017); this means the color of *A. arborescens* was lighter compared to *A. abyssinica*. *Artemisia arborescens* also had higher a* and b* values, indicating a tendency toward redness and yellowness. The total color difference (ΔE*) was higher in *A. abyssinica* (37.75 ± 0.03) compared to *A. arborescens* (37.14 ± 0.00). Similar results were found with *C. rotundifolia* leaves by Al‐Bukhaiti et al. (2019), and they mentioned that the color of leaf powder is influenced by the raw material's composition, such as carbohydrates and proteins. Furthermore, the discrepancies in the color features of the examined plants might be attributed to variances in pigments such as chlorophyll, which rely on the botanical origin as well as the structure composition (Mohammed et al., 2019). Moreover, Preethi et al. (2022) noted that the presence of reducing sugars and amino acids could impact the color of samples, which aligns with the findings on sugar content and amino acid composition in our study. Additionally, the presence of phenolics and other constituents may contribute to the higher L* value observed, while lower feed moisture levels could affect the a* value (Pandiselvam et al., 2023). These assertions are further corroborated by the TPC values obtained in this study.

Table 1 indicates the pH values of the *A. abyssinica* and *A. arborescens* leaves (5.67 ± 0.00 and 5.64 ± 0.00, respectively), with significant differences (*p* < .05) observed. These results were comparable with the results of *Malva neglecta* (5.27–6.55), *P. cognatum* (5.76–6.57), and *T. orientalis* (5.28–6.18) (Kibar & Kibar, 2017) and were higher than the pH value of *the C. rotundifolia* leaves (4.53), reported by Al‐Bukhaiti et al. (2019), who stated that the chemical composition of sugars and fatty acids in plants provides a distinct variation that contributes to plant acidity variability. The grinding process is another factor that can significantly affect leaf powder pH measurements due to the resultant particle size, with more extensive physical damage and oxidation of leaf samples leading to higher pH values (Chen et al., 2022). Moreover, the degradation of chemical compounds within plant samples can influence pH values. Almutairi et al. (2023) noted that the hydrolysis of salts like Na, K, Mg, and Ca in raw materials might increase pH levels. Also, soil fertilization practices can induce soil acidification or alkalinization, producing pH disparities among plant samples (Courchesne et al., 1995).

Nevertheless, the acidity levels observed in the studied plants are likely suitable for their utilization as industrial supplements, posing minimal risk to either food properties or consumers. Remedio et al. (2023) indicated that pH values falling within the range of 5.8–7.1 do not irritate the oral mucosa. Furthermore, plant powder with a moderate pH plays a pivotal role in food applications. Wang et al. (2019) underscored the significance of pH in controlling chemical reactions during processing and fermentation, as well as regulating microbial growth. Additionally, adjusting the pH of plant powders can influence their gelling ability, foaming properties, emulsification, solubility, and WAC (Raikos et al., 2014). Pacifico et al. (2021) highlighted the development of plant powders from Brassicaceae family crops for food and plant protection purposes, where pH likely influences their efficacy as herbicides and insecticides.

### 3.3 Functional properties

#### 3.3.1 WAC and OAC

**FIGURE 2:**
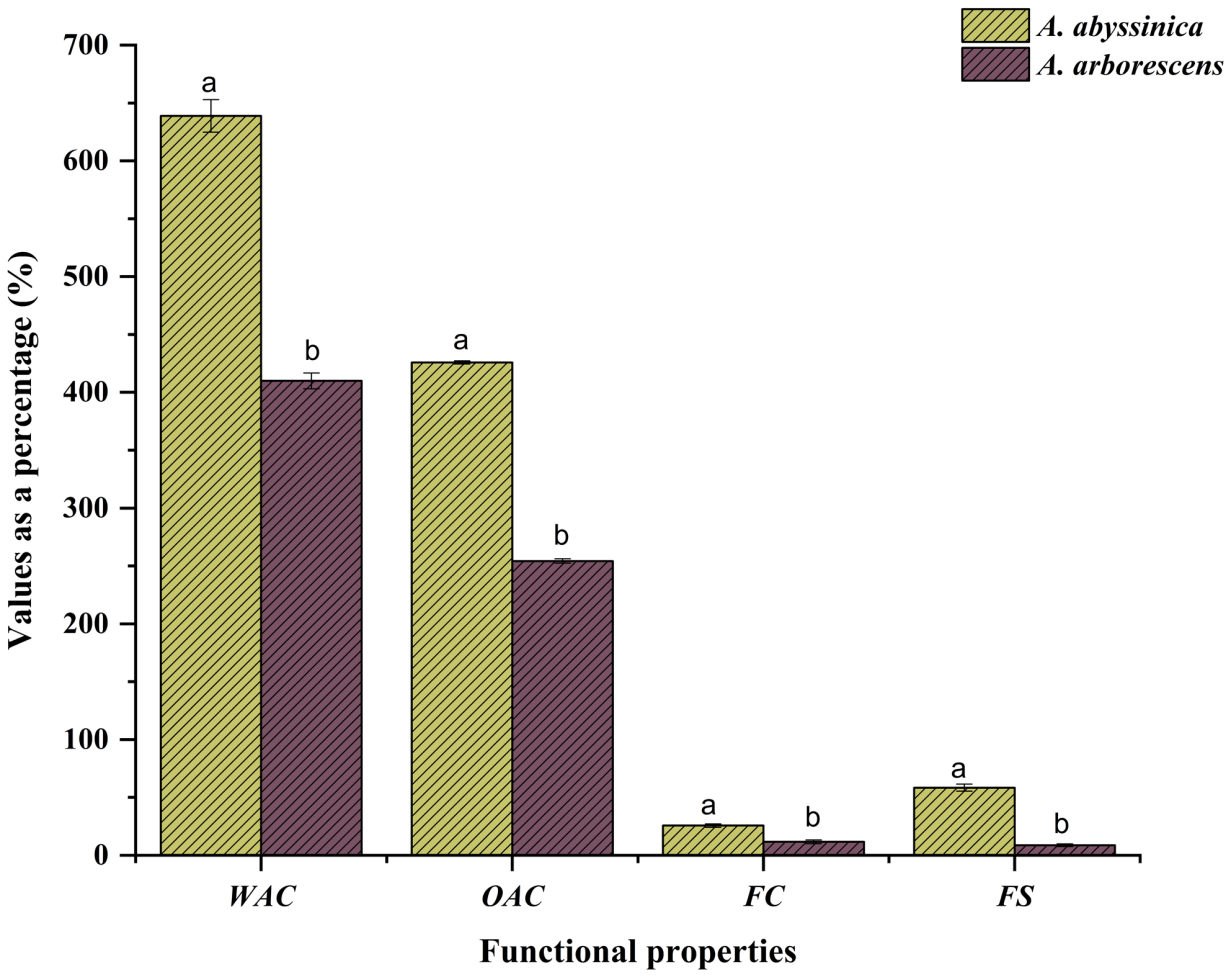
Functional properties of *Artemisia abyssinica* and *Artemisia arborescens* plant powders. FC, foaming capacity; FS, foaming stability; OAC, oil absorption capacity; WAC, water absorption capacity. The different letters indicate that the values differ substantially (*p* < .05).

The functional properties of *A. abyssinica* and *A. arborescens* leaf powders were assessed in terms of their WAC and OAC, as illustrated in Figure 2. Water absorption properties reflect a product's ability to bind with water when water is limited (Tsehay et al., 2023). *Artemisia abyssinica* leaf powder displayed a WAC of 638.81 ± 34.11%, while *A. arborescens* leaf powder showed a WAC of 409.98 ± 6.83%. In terms of OAC, *A. abyssinica* had an OAC of 425.85 ± 1.33%, while *A. arborescens* had an OAC of 254.26 ± 1.93%. Comparatively, these powders exhibited higher WAC and OAC values than those observed in Argun fruit powder (kernels, flesh, and peel), as studied by Mohammed et al. (2019), as well as in *C. rotundifolia* leaf powder, as investigated by Al‐Bukhaiti et al. (2019). The disparity between WAC and OAC may be attributed to the distinct chemical compositions of the two plants. The elevated WAC of *A. abyssinica* may be attributed to the existence of distinct hydrophilic carbohydrates or proteins that have an affinity for water molecules, therefore attracting them. Pandiselvam et al. (2023) stated that the existence of proteins and carbohydrates, which typically provide significant hydrogen bonding because of their polar or charged side chains, can influence WAC. Moreover, the authors postulated that the increase in WAC may be attributed to the particle size, as larger particle sizes tend to decrease WAC values. In addition, the presence of negative charges on the phosphate groups in some plant compounds enhances their capacity to bind water. Conversely, Sotelo‐Díaz et al. (2023) found that the hydroxyl groups had a higher likelihood of making hydrogen bonds with water, which leads to an increase in the WAC in the material containing these groups. Furthermore, the authors noted that the presence of protein in the mixtures could potentially lead to a reduction in OAC. According to Shameena Beegum et al. (2019), there may be a relationship between protein denaturation, changes in starch structure, and the rise in WAC. The authors suggested that changes in the structure caused by protein denaturation decreased solubility, increasing the protein's hydrophilic nature due to its interaction with starch through cross‐linking. These explanations can be related to the crude proteins and monosaccharides in Table 1. On the other hand, González‐Jiménez et al. (2022) claimed that the elevated OAC in the samples had a positive association with the lipid content in the samples. The protein composition of the sample has a profound impact on the characteristics of OAC. This is because the polar amino acids' side chains can form hydrophobic interactions with the hydrocarbon chains of lipids (Al‐Bukhaiti et al., 2019). The findings of the total fat content in Table 1 and the fatty acids in Table 5 obtained in this investigation were in line with this conclusion.

However, these findings indicate that powders with higher WAC could be employed as thickeners in liquid and semiliquid foods. This is because they can absorb water and swell, enhancing the consistency of food products (Tsehay et al., 2023). So, *A. abyssinica* powder, with its strong WAC, is particularly suitable as a raw material in bakery products and various other applications. Furthermore, the capacity of a food component to effectively capture oil through a complex capillary attraction mechanism is crucial in various applications. This attribute of powder contributes to improved mouthfeel, consistency, and flavor retention (Nwajagu et al., 2021). So, the OAC plays a significant role in food formulations by enhancing flavor and mouthfeel.

#### 3.3.2 FC and FS

Foam formation is crucial in the baking industry, especially for products like cakes and biscuits. During the whipping process, proteins accumulate because they spread out over a larger area at the interface between the liquid and air. However, the FC and FS of *A. abyssinica* and *A. arborescens* leaf powders were measured, and the results are presented in Figure 2. As shown, the FC and FS of *A. abyssinica* leaf powder (25.67 ± 1.53% and 58.48 ± 2.99%, respectively) were higher than those observed with *A. arborescens* (11.67 ± 1.53% and 8.68 ± 1.19%, respectively). These results were somewhat similar to the FC of *C. rotundifolia* leaf powder (14%) reported by Al‐Bukhaiti et al. (2019), but notably higher than that of acacia seed powder (7.17%), as reported by Embaby and Rayan (2016). The high FC might be attributed to its increased protein content in the tested plants, which enhances its foaming properties (Elkhalifa & Bernhardt, 2010). Additionally, the presence of other compounds in the hydrocolloid, along with differences in structure and molecular weight, can also influence foaming properties, as suggested by Wang et al. (2018). Moreover, increased carbohydrate content can improve the ability to form foam. According to Donatus et al. (2023), the presence of sucrose considerably enhances the foaming potential. So, the results in this investigation were consistent with the sucrose finding, as shown in Table 1. Another possible cause is that the plant cells of *A. abyssinica* are more susceptible to disruption, resulting in a greater amount of air being integrated into the foam. Based on the findings of this study, it appears that *A. abyssinica* is a more suitable option than *A. arborescens* for situations that require foaming, such as foamed beverages, foamed insulation, foamed cleaning solutions, as well as cosmetics or food‐related products.

### 3.4 Volatile composition

**FIGURE 3:**
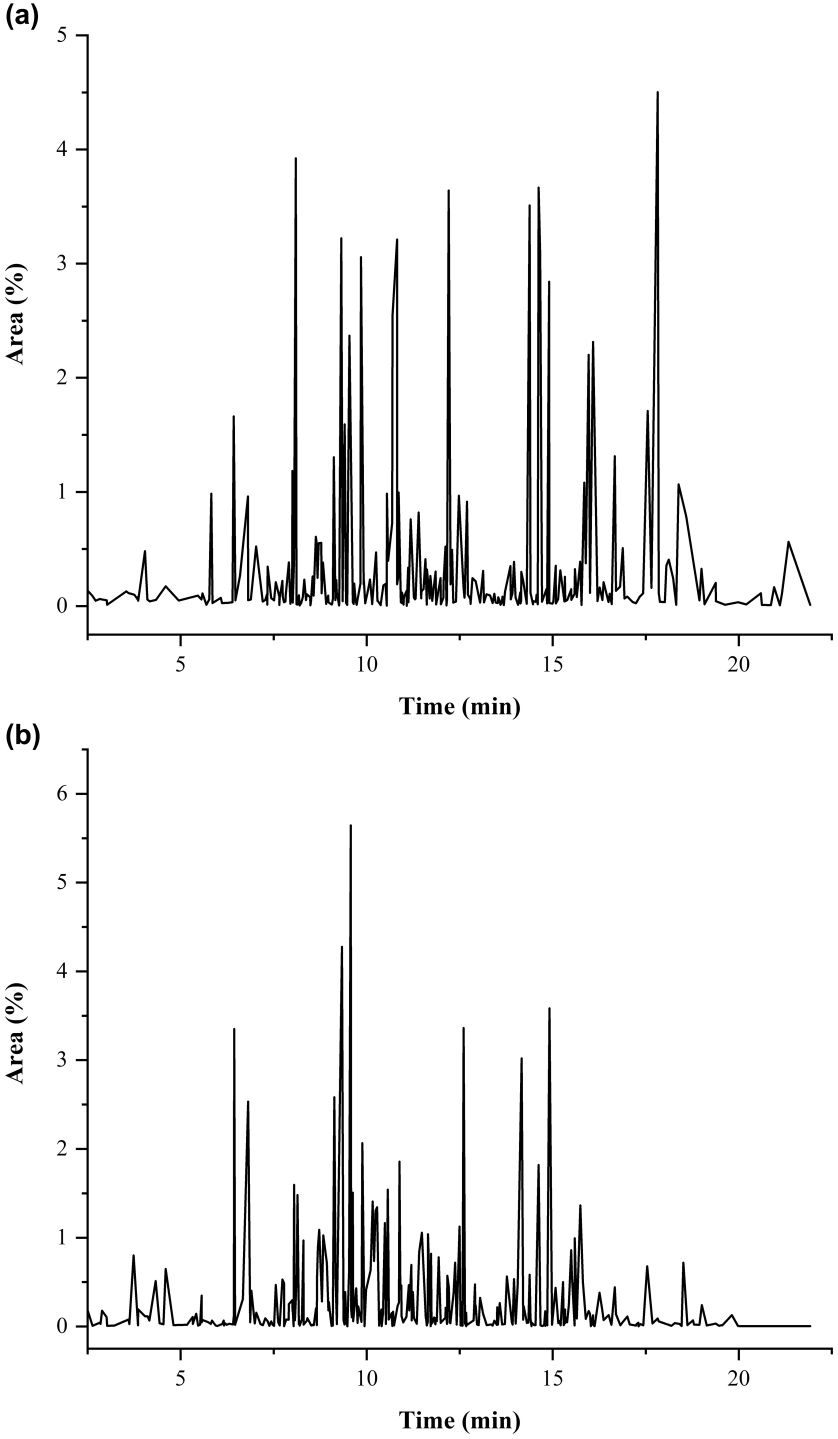
Chromatogram of detected volatile composition in *Artemisia abyssinica* (a) and *Artemisia arborescens* (b) plant powders as analyzed using GC–MS.

The analysis of volatile compounds in *A. abyssinica* and *A. arborescens* leaf powders was conducted using GC–MS, and the results are summarized in Table 2 and Figure 3. In *A. abyssinica*, a total of 307 compounds were identified, with concentrations ranging from 6.21% to 0.003%, while a total of 331 volatile compounds were identified in *A. arborescens*, with concentrations ranging from 8.08% to 0.001%. These compounds play a significant role in the aroma and potential health benefits of these plants. Notable compounds in *A. abyssinica* include (2aS,3aR,5aS,9bR)‐2a,5a,9‐trimethyl‐2a,4,5,5a,6,7,8,9b‐octahydro‐2H‐naphtho[1,2‐b]oxireno[2,3‐c]furan (6.21%), which is known for its distinctive aroma, followed by bicyclo[3.1.1]heptan‐3‐one, 6,6‐dimethyl‐2‐(2‐methylpropyl) (5.59%), and hotrienol (4.53%). In contrast, the most abundant compound in *A. arborescens* was artemisia alcohol (8.08%), followed by butanoic acid, 3‐hexenyl ester, (E)‐ (4.28%), and (E)‐β‐Famesene (3.58%), which impart distinct aromas and may have health‐related properties. These findings are in line with those of Asfaw and Demissew (2015), who found that the main constituents in the EOs of *A. abyssinica* were Yomogi alcohol (37.6%), artemisyl acetate (22.4%), and artemisia alcohol (8.8%). Additionally, Tariku et al. (2010) discovered that Yomogi alcohol (38.47%), artemisyl acetate (24.88%), artemisia alcohol (6.70%), santolina triene (1.78%), and 1,8‐cineole (1.56%) were the main volatile components of *A. abyssinica* EOs. As reported by Said et al. (2016), various EOs displayed a comparable terpene composition, primarily containing chamazulene, β‐thujone, and camphor. Nevertheless, the specific levels of these compounds differed among the EOs derived from the aerial parts of *A. arborescens*, depending on the geographical location in the Mediterranean area where they were collected. According to the phytochemical analysis of the EOs found in the leaves of *A. arborescens* by Riahi et al. (2022), the primary components were chamazulene (45.01%–47.02%), camphor (24.94%–25.75%), β‐myrcene (5.52%–5.64%), and germacrene D (4.30%–4.72%). The authors also pointed out that the mother plant contained the EOs components α‐copaene, terpinen‐4‐ol, α‐terpinene, and α‐thujene. However, other *Artemisia* species could contain unique EOs that make up their primary constituents. For instance, the main components of the EOs of *Artemisia campestris*, *Artemisia herba‐alba*, and *Artemisia absinthium* were α‐thujone (29.39%), chamazulene (39.21%), and β‐pinene (32.07%), respectively (Umam et al., 2023).

**TABLE 2:** Chemical composition of volatile compounds of *Artemisia abyssinica* and *Artemisia arborescens* plant powders.

*A. abyssinica*			*A. arborescens*		
Name	R.T.	Area %	Name	R.T.	Area %
2aS,3aR,5aS,9bR)‐2a,5a,9‐Trimethyl‐2a,4,5,5a,6,7,8,9b‐octahydro‐2H‐naphtho[1,2‐b]oxireno[2,3‐c]furan	17.82	6.21	Artemisia alcohol	9.57	8.08
Bicyclo[3.1.1]heptan‐3‐one, 6,6‐dimethyl‐2‐(2‐methylpropyl)‐	16.08	5.59	Butanoic acid, 3‐hexenyl ester, (E)‐	9.34	4.28
Hotrienol	9.32	4.53	(E)‐β‐Famesene	14.91	3.58
Yomogi alcohol	8.1	3.92	Lavandulyl acetate	12.6	3.36
Caryophyllene	14.62	3.67	Santolina triene	6.44	3.35
Carvotanacetone	12.21	3.64	Artemisyl acetate	10.88	3.26
Thymohydroquinone dimethyl Ether	14.38	3.51	(−)‐*Cis*‐Beta‐Elemene	14.16	3.02
Artemisyl acetate	10.82	3.21	Hotrienol	9.13	2.58
2,5‐Dimethoxy‐4‐isopropenyltoluene	14.66	3.09	Artemisia triene	6.81	2.53
Linalool	9.85	3.06	Yomogi alcohol	8.14	2.09
(E)‐β‐Famesene	14.9	2.84	Linalool	9.88	2.07
Camphor	10.7	2.55	Caryophyllene	14.62	1.82
Artemisia alcohol	9.53	2.37	Alpha‐ocimene	8.05	1.60
Santolina triene	6.43	1.66	2,6‐Dimethyl‐2,6‐octadiene‐1,8‐diol diacetate	11.73	1.60
Sabinene hydrate	9.41	1.59	3‐Methyl‐3‐nitrobut‐1‐ene	10.56	1.54
Caryophyllene oxide	16.67	1.31	2,3‐Dimethyl‐1‐hexene	10.16	1.41
Heptane, 2,2,4,6,6‐pentamethyl‐	8.01	1.18	4,6‐Heptadien‐2‐one, 3,6‐dimethyl‐3‐(1‐methylethyl)‐, (E)‐	15.74	1.36
6,6‐Dimethyl‐cyclooct‐4‐enone	18.38	1.07	(E)‐4,8‐Dimethylnona‐1,3,7‐triene	10.28	1.34
Lavandulol	10.86	1.00	Dicyclopropyl carbinol	10.49	1.16
1,7‐Nonadien‐4‐ol, 4,8‐dimethyl‐	10.55	0.99	Cyclopropanemethanol, 2,2‐dimethyl‐3‐(2‐methylpropenyl)‐, acetate, trans	12.49	1.13
Hexyl alcohol	5.83	0.99	2,7‐Dimethyl‐4(E), 6‐octadien‐2‐Ol	8.72	1.09
Eicosane	12.48	0.97	Pentane, 1‐(2‐butenyloxy)‐, (E)‐	11.48	1.06
Artemisia triene	6.81	0.96	2‐Hexanone, 3‐methyl‐	11.65	1.04
Acetic acid	1.98	0.92	Lavender lactone	8.83	1.03
Bornyl acetate	12.7	0.91	Naphthalene, 1,2,3,4,4a,5,6,8a‐octahydro‐4a,8‐dimethyl‐2‐(1‐methylethenyl)‐, [2R‐(2α,4aα,8aβ)]‐	15.59	0.99
α‐Terpineol	11.4	0.82	(3E,5E)‐2,6‐dimethylocta‐3,5,7‐trien‐2‐ol	8.3	0.97
3,5‐Heptadien‐2‐one, 6‐methyl‐	9.91	0.81	Ketone, 1,5‐dimethylbicyclo[2.1.0]pent‐5‐yl methyl	8.69	0.89
Camphenol	18.59	0.79	(2,6,6‐Trimethylcyclohex‐1‐enylmethanesulfonyl)benzene	15.49	0.86
4‐Carvomenthenol	11.18	0.76	α‐Terpineol	11.41	0.84
(+)‐Camphor	10.69	0.73	Pyridine	3.74	0.80

In addition, it was found that both plants contained comparable volatile substances in different quantities, including artemisyl acetate, yomogi alcohol, α‐terpineol, linalool, and caryophyllene. These compounds offer various benefits. α‐Terpineol, found in the EOs of plants, exhibits anticancer, antimicrobial, anti‐inflammatory, analgesic, antioxidant, cardioprotective, neuroprotective, gastroprotective, and antidiarrheal effects, making it versatile for food, medical, cosmetic, and agricultural industries (Chen, Zhang, et al., 2023). The Yomogi oil extracted from *Artemisia montana* (Yomogi) contains significant amounts of camphor, borneol, α‐piperitone, and caryophyllene oxide. It possesses calming qualities and has the ability to reduce stress and salivary α‐amylase activity (Kunihiro et al., 2017). Linalool, a common terpene, serves as an anxiolytic, sedative, antistress, antidepressant, antimicrobial, anticancer, anti‐inflammatory, hepatoprotective, renal protective, and neuroprotective agent and supports the processes that support the protective effects on the lungs (An et al., 2021). Caryophyllene, present in EOs, acts as an anti‐inflammatory and analgesic agent (Gyrdymova & Rubtsova, 2022).

### 3.5 Amino acid analysis

Amino acids are the building blocks of proteins and play a crucial role in human nutrition and various physiological functions. Table 3 provides a detailed comparison of the amino acid composition of raw plant powders from *A. abyssinica* and *A. arborescens*, with the values expressed in mg/100 g of DW. The recommended dietary intake (RDI) and the percentage contribution to RDI (% C‐RDI) for each amino acid were also compared. A total of 18 free amino acids were identified and quantified in the analyzed samples. *Artemisia arborescens* exhibited a higher total amino acid content (15,478.36 ± 7.52 mg/100 g DW) than *A. abyssinica* (14,715.92 ± 4.08 mg/100 g DW). The findings also revealed that both *A. abyssinica* and *A. arborescens* plants contain eight essential amino acids, with leucine being the most abundant, which reached 1061.53 mg/100 g DW in *A. abyssinica*, whereas it was slightly higher in *A. arborescens* (1082.08 mg/100 g DW), followed by valine with a value of 878.96 mg/100 g DW and 1054.71 mg/100 g DW, respectively. Essential amino acids offer various benefits across different health aspects. Martínez Sanz et al. (2019) claimed that essential amino acids, especially branched chain amino acids (leucine, valine, and isoleucine), are essential for muscle recovery, protein synthesis stimulation, and fatigue reduction. Also, essential amino acids play a crucial role in attenuating muscle loss post total knee arthroplasty, aiding in muscle volume preservation and functional mobility improvement in older adults. In aging bone, essential amino acids contribute positively by potentially increasing bone mass, promoting osteoblast proliferation, and decreasing osteoclast activity (Ismail Pandor et al., 2023). Moreover, amino acids have been proven to be crucial in decreasing oxidative stress, functioning as antioxidants and anti‐inflammatory agents, and are utilized to improve sleep and mood disorders, enhance exercise performance, prevent muscle loss, and facilitate weight loss (Bisht et al., 2022). Additionally, essential amino acid mixtures show promise in managing behavioral and psychological symptoms of dementia in Alzheimer's patients (Takada et al., 2022).

**TABLE 3:** Essential and nonessential amino acids compositions (mg/100 g DW) of *Artemisia abyssinica* and *Artemisia arborescens* plant powders.

	Amino acid content mg/100 g DW		RDI (mg)	% C‐RDI	
	*A. abyssinica*	*A. arborescens*		*A. abyssinica*	*A. arborescens*
Essential amino acids					
Histidine [His]	326.17 ± 1.02^a^	323.02 ± 0.82^a^	700	46.60	46.15
Isoleucine [Ile]	656.11 ± 1.41^b^	726.56 ± 3.66^a^	1400	46.87	51.90
Lysine [Lys]	694.34 ± 2.32^a^	669.74 ± 4.45^b^	2100	33.06	31.89
Leucine [Leu]	1061.53 ± 6.09^a^	1082.08 ± 20.57^a^	2730	38.88	39.64
Methionine [Met]	190.39 ± 0.98^a^	108.84 ± 1.22 ^b^	728	26.15	14.95
Phenylalanine [Phe]	811.58 ± 0.93^a^	763.70 ± 2.09^b^	1750	46.38	43.64
Threonine [Thr]	599.91 ± 1.75^a^	550.13 ± 1.76^b^	1050	57.13	52.39
Valine [Val]	878.96 ± 3.52^b^	1054.71 ± 12.52^a^	1820	48.29	57.95
Nonessential amino acids					
Alanine [Ala]	788.53 ± 2.12^a^	738.3 ± 4.09^b^	–	–	–
Arginine [Arg]	1147.68 ± 1.08^a^	907.65 ± 0.82^b^	–	–	–
Aspartic acid [Asp]	2492.63 ± 6.07^b^	2791.56 ± 11.92^a^	–	–	–
Cysteine [Cys‐s]	109.88 ± 0.75^a^	195.34 ± 0.94^a^	280	39.24	69.76
Glycine [Gly]	725.77 ± 2.03^a^	713.69 ± 0.81^b^	–	–	–
Glutamic [Glu]	1771.07 ± 5.18^b^	2286.07 ± 13.09^a^	–	–	–
Proline [Pro]	1436.63 ± 4.01^b^	1534.74 ± 9.03^a^	–	–	–
Serine [Ser]	555.99 ± 0.98^b^	611.24 ± 1.68^a^	–	–	–
Tyrosine [Tyr]	468.76 ± 0.03^a^	420.99 ± 1.01^b^	–	–	–
Total	14,715.92 ± 4.08^a^	15,478.36 ± 7.52^a^			

*Note*: Different letters show significant changes (*p* < .05) in the same line; values are means ± standard deviation of three determinations.

In the same context, both plants exhibit the presence of eight nonessential amino acids, with aspartic acid being the predominant one, which reached 2492.63 mg/100 g DW in *A. abyssinica*, while it was 2791.56 mg/100 g DW in *A. arborescens*. Furthermore, glutamic acid is another notable nonessential amino acid found in both plants, with 1771.07 mg/100 g DW in *A. abyssinica* and 2286.07 mg/100 g DW in *A. arborescens*. Nonessential amino acids offer various benefits to human health. Aspartic acid has been utilized in inhibiting the formation of advanced glycation end products, which are implicated in various diseases, showcasing its potential in preventing complications caused by glycation (Prasanna & Saraswathi, 2016). Glutamic acid serves as the primary excitatory neurotransmitter, exerting its effects in both the brain and peripheral tissues, particularly in skin health and regeneration, as well as promoting hair growth, keratinocyte proliferation, and skin apoptosis, indicating its potential in treating skin conditions like psoriasis (Jara et al., 2021). Glycine, a crucial nonessential amino acid, promotes growth, health, and metabolic processes by serving as a precursor for vital metabolites. Also, supplementing the diet with an appropriate quantity of glycine has proven effective in treating metabolic abnormalities in individuals with cardiovascular diseases, diabetes, cancer, obesity, and inflammatory conditions (Razak et al., 2017). In addition, a combination of six nonessential amino acids has been shown to improve joint conditions, reducing pain, discomfort, and stiffness, thus enhancing the quality of life (Takeuchi et al., 2022). Furthermore, nonessential amino acids like serine and glycine play a critical role in proliferative metabolism and can inhibit tumor growth under specific dietary conditions (Sullivan & Vander Heiden, 2017). These amino acids also have significant roles in cancer metabolism, serving as precursors for macromolecules, controlling redox status, and being targets for cancer therapy development (Choi & Coloff, 2019).

However, both species contributed varying percentages of the RDI for essential and nonessential amino acids. These findings resembled those of Adetunji et al. (2023), who reported leucine (770 mg/100 g) and valine (870 mg/100 g) as prominent essential amino acids in *Evolvulus alsinoides*. They also identified aspartic acid (1002 mg/100 g) and glutamic acid (1160 mg/100 g) as the most abundant among the nonessential amino acids (5330 mg/100 g). However, such a divergence in the amino acid concentration may be impacted by geographical factors, ecological, biological, and plant species, as well as the product's handling and storage circumstances (Urcan et al., 2021). Furthermore, the presence of protein in the samples could be a significant factor in elevating the levels of amino acids within those samples. This hypothesis finds support in the data presented in Table 1, which displays the protein content.

### 3.6 Mineral element analysis

Table 4 presents the mineral composition (mg/100 g DW) of *A. abyssinica* and *A. arborescens* plant powders. The study results revealed that the examined plants included several types of micro‐ and macroelements essential for treating many different diseases. However, *A. arborescens* has a slight edge over *A. abyssinica* in terms of mineral content, as it is a better source of magnesium (90.04 ± 0.22 mg/100 g DW), manganese (2.37 ± 0.03 mg/100 g DW), iron (72.68 ± 0.20 mg/100 g DW), and copper (0.20 ± 0.01 mg/100 g DW) than *A. abyssinica*, while *A. abyssinica* outperformed *A. arborescens* in sodium (31.46 ± 1.02 mg/100 g DW), calcium (238.07 ± 0.09 mg/100 g DW), and zinc (0.52 ± 0.01 mg/100 g DW).

**TABLE 4:** Mineral composition (mg/100 g DW) of *Artemisia abyssinica* and *Artemisia arborescens* plant powders.

	Mineral composition mg/100 g DW		RDI (mg)	% C‐RDI	
	*A. abyssinica*	*A. arborescens*		*A. abyssinica*	*A. arborescens*
Sodium [Na]	31.46 ± 1.02^a^	2.17 ± 0.08^b^	2300 _UL_	1.37	0.09
Magnesium [Mg]	80.73 ± 0.19^b^	90.04 ± 0.22^a^	420 _RDA_	19.22	21.44
Potassium [K]	440.91 ± 1.23^a^	440.20 ± 5.01^a^	4700 _AI_	9.38	9.37
Calcium [Ca]	238.07 ± 0.09^a^	199.68 ± 0.02^b^	1000 _RDA_	23.81	19.968
Manganese [Mn]	1.49 ± 0.01^b^	2.37 ± 0.03^a^	2.3 _AI_	64.78	103.04
Iron [Fe]	36.19 ± 0.12^b^	72.68 ± 0.20^a^	8 _RDA_	452.38	908.50
Cobalt [Co]	0.01 ± 0.00^a^	0.03 ± 0.00^a^	–	–	–
Nickel [Ni]	0.07 ± 0.00^b^	0.15 ± 0.00^a^	–	–	–
Copper [Cu]	0.12 ± 0.00^b^	0.20 ± 0.01^a^	0.9 _RDA_	13.33	22.22
Zinc [Zn]	0.52 ± 0.01^a^	0.33 ± 0.01^b^	11 _RDA_	4.73	3.00

*Note*: Different letters show significant changes (*p* < .05) in the same line; values are means ± standard deviation of three determinations.

Abbreviations: AI, adequate intake; RDA, recommended dietary allowance; UL, the tolerable upper intake level.

According to Bhupathyraaj et al. (2024), sodium is the primary positively charged ion in the area outside of cells. It is essential for the proper functioning of the human body and is found in various types of food. Sodium ions are necessary to regulate blood and body fluids, transmit nerve impulses, maintain heart activity, and support certain metabolic functions. Magnesium, which is the fourth most common mineral in human bodies, is a crucial cofactor in more than 300 metabolic enzymatic processes and is involved in several stages of both the insulin‐signaling pathways and glucose metabolism (Cao et al., 2023). Magnesium deficiency is a recognized health concern and can contribute to various conditions, including cardiovascular issues and connective tissue disorders (Sankova et al., 2024). On the other hand, manganese controls neurotransmitter activation in neuromuscular diseases, whereas iron plays an important part in deoxyribonucleic acid (DNA) synthesis by activating several enzymes involved in brain neurotransmitters; copper serves as a crucial cofactor for various enzymes, assisting in vitamin C oxidation and supporting hemoglobin production alongside iron; and zinc is required for glucose metabolism and DNA synthesis (Mayuri et al., 2022). Calcium is a crucial element that the body cannot produce and must be obtained from external sources, primarily through food. It is related to mental illnesses and serves as an activator for several enzymes in the body, facilitating the proper functioning of organs (Shen et al., 2023). On the other hand, iron, copper, and zinc are trace elements that are necessary for the optimal functioning of living organisms and play a role in various processes, such as anti‐inflammatory, antioxidant, cellular metabolism, controlling gene expression, enzyme activity, contributing to protein synthesis, and having a significant impact on the health of pregnant women, fetal development, and the health of newborns (Grzeszczak et al., 2020).

Also, both plants provide essential minerals, though their concentrations may vary, potentially offering complementary contributions to overall mineral intake in the diet. Nevertheless, these plants had a comparable higher content of most mineral compositions than other root and tuber crops such as yam, cassava, and sweet potato, as well as the raw flesh and skin of red and white potatoes (Chandrasekara & Josheph Kumar, 2016). It was also higher than most mineral compositions found in Hambaguyta and Bagana tuber flours (Tsehay et al., 2023) and the pulp and peel of *Citrus medica* (Mahdi, Al‐Ansi, et al., 2020).

### 3.7 Organic acid compositions

**FIGURE 4:**
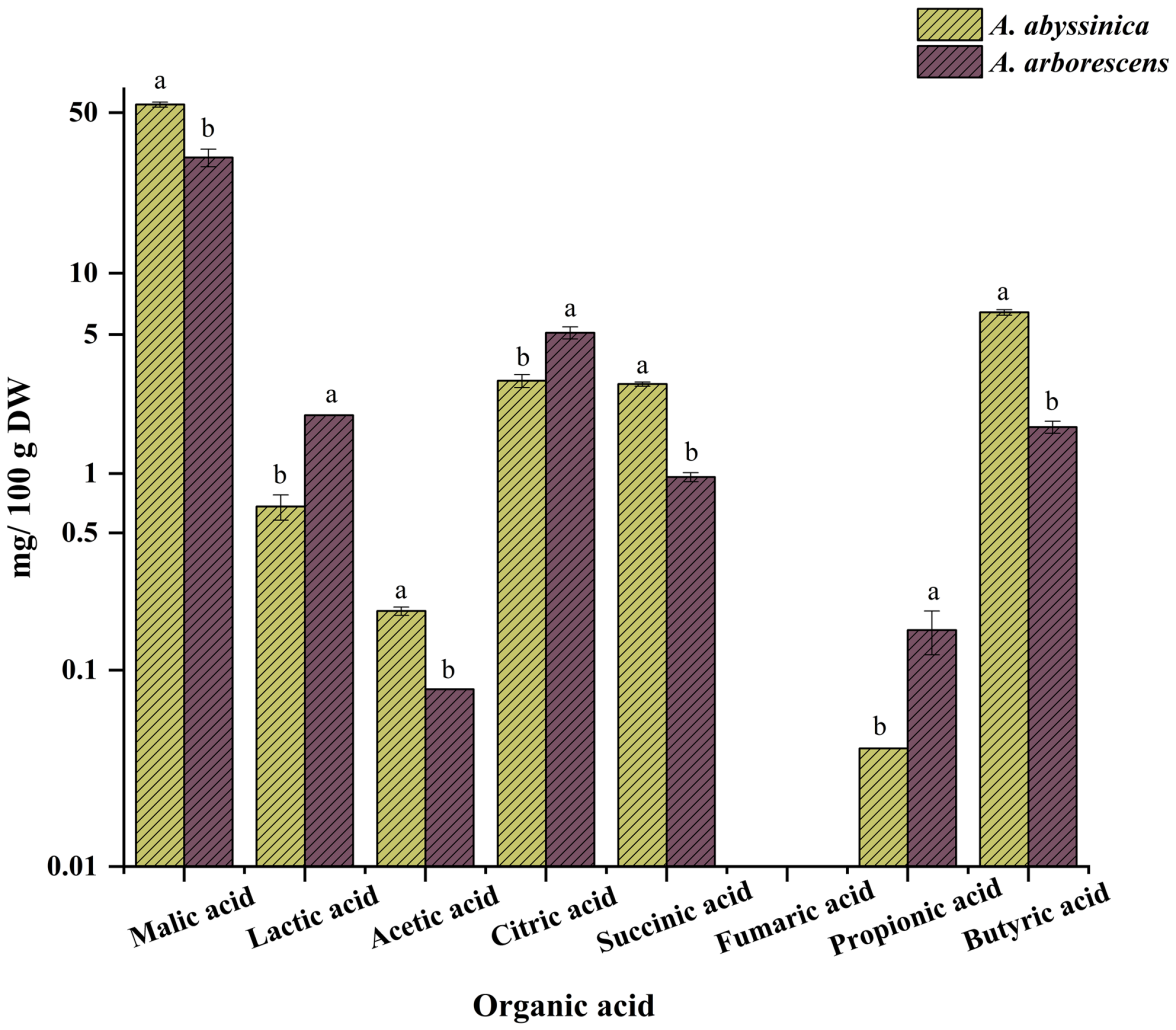
Organic acid composition (mg/100 g DW) of *Artemisia abyssinica* and *Artemisia arborescens*. The different letters indicate that the values differ substantially (*p* < .05).

The organic acid content of the examined plant is detailed in Figure 4. Eight organic acids were identified in both plants. Notably, *A. abyssinica* had higher levels of malic acid (53.33 ± 1.02 mg/100 g DW), acetic acid (0.2 ± 0.01 mg/100 g DW), succinic acid (2.83 ± 0.07 mg/100 g DW), and butyric acid (6.44 ± 0.2 mg/100 g DW) compared to *A. arborescens*. Conversely, *A. arborescens* exhibited higher amounts of lactic acid (1.97 ± 0.01 mg/100 g DW), citric acid (5.11 ± 0.35 mg/100 g DW), fumaric acid (0.004 ± 0.00 mg/100 g DW), and propionic acid (0.16 ± 0.04 mg/100 g DW) compared to *A. abyssinica*. However, it is essential to note that the specific composition of these organic acids varied significantly based on the plant part and the harvesting stage (Petropoulos et al., 2019). Chen, Yu, et al. (2023) and Chen, Zhang, et al. (2023) claimed that organic acids constitute around 3% of the total weight of tea leaves, and their composition and quantities fluctuate across various tea varieties. Similarly, Oliveira et al. (2009) investigated organic acid content in the leaves and stems of different *Portulaca oleraceae* genotypes and found notable distinctions in organic acid levels between various plant parts, though the extent of these differences depended on the genotype under scrutiny. Szalai et al. (2010) observed the presence of oxalic and malic in the leaves of three *Portulaca oleraceae* species, and the harvesting stage primarily influenced these variations. These findings underscore the variability in organic acid composition within plants, emphasizing the importance of considering both the plant part and harvesting stage in assessing organic acid profiles.

Measuring the levels of organic acids in various food types enables us to track the sensory characteristics, determine the origin, and ensure quality control. Additionally, this information serves as a foundation for designing food formulations, enhancing flavor, preserving nutritional value, and prolonging the shelf life of food (Shi et al., 2022). Organic acids also provide health benefits, including antioxidation, enhancement of digestion and absorption, acceleration of gastrointestinal transit, and management of intestinal flora (Chen, Yu, et al., 2023), as well as reducing inflammation, preventing osteoporosis, regulating the immune system, producing intestinal hormones, reducing obesity, promoting calcium absorption, and preventing blood clotting (Ji et al., 2021). Moreover, organic acids function as antifungal and anticoccidial agents, impeding the proliferation of harmful organisms. Appropriate dosages of organic acids enhance growth, improve health conditions, and reduce mortality rates (Bansod et al., 2020).

### 3.8 Fatty acid compositions

Fatty acids conduct various vital biological functions in vivo (Carvalho et al., 2011). Table 5 lists the fatty acids of *A. abyssinica* and *A. arborescens*. *Artemisia abyssinica* had 14 fatty acids identified, with concentrations ranging from 61.11% to 0.16%, whereas *A. arborescens* had 13 fatty acids identified, with concentrations ranging from 56.75% to 0.17%. Palmitic acid (C16:0) as saturated fatty acids (SFAs) and linoleic acid (C18:2) as unsaturated fatty acids (USFAs) were the most abundant components in both plants, accounting for 61.11% and 8.80% in *A. abyssinica*, respectively, and 59.75% and 15.30% in *A. arborescens*. Furthermore, it was discovered that both plants contained equivalent fatty acids in varying amounts, such as linolenic acid (C18:3), lauric acid (C12:0), and nonanoic acid (C9:0). Albakry et al. (2023) reported similar results in *Nigella sativa* oils, discovering that C18:2 and C18:1 were important USFAs in black seed oil, whereas C16:0 was the dominant SFA.

**TABLE 5:** Chemical composition of fatty acids of raw *Artemisia abyssinica and Artemisia arborescens* plants.

*A. abyssinica*			*A. arborescens*		
Name of fatty acids	R.T.	Area %	Name of fatty acids	R.T.	Area %
Saturated fatty acids					
Palmitic acid (C16:0)	7.86	61.11	Palmitic acid (C16:0)	7.86	59.75
Tetradecanoic acid, 12‐methyl‐, (C15:0)	6.21	8.04	Tetradecanoic acid (C14:0)	5.38	2.42
Pentadecanoic acid (C16:0)	6.11	1.29	Heptacosanoic acid, 25‐methyl‐ (C26:0)	17.94	1.61
Margaric acid (C17:0)	8.78	0.97	Tetradecanoic acid, 12‐methyl‐(C15:0)	6.21	0.48
Eicosanoic acid (C20:0)	14.78	0.89	Arachidic acid (C20:0)	14.78	0.41
Hexacosanoic acid (C26:0)	21.52	0.57	Lauric acid (C12:0)	3.53	0.35
Lauric acid (C12:0)	3.53	0.42	Nonanoic acid (C9:0)	2.08	0.26
Elaidic acid (C18:1, Trans)	10.69	0.27	Pentadecanoic acid (C15:0)	6.56	0.20
Nonanoic acid (C9:0)	2.08	0.23	Margaric acid (C17:0)	9.35	0.17
Tetradecanoic acid (C14:0)	4.99	0.16			
Unsaturated fatty acids					
Linoleic acid (C18:2)	10.48	8.80	Linoleic acid (C18:2)	10.48	15.30
Oleic acid (C18:1, cis)	10.59	4.67	Linolenic acid (C18:3)	10.58	7.20
Linolenic acid (C18:3)	10.58	3.69	Trans‐13‐Octadecenoic acid (C18:1, Trans)	10.60	2.78
7‐Hexadecenoic acid (C16:1)	7.55	0.75	Lignoceric acid (C24:0)	19.00	0.49

Fatty acids are essential for various bodily functions, including signaling, energy storage, and membrane structure regulation (Mumtaz et al., 2020). Palmitic acid, a saturated fatty acid, has been extensively studied for its impact on health. Research suggests that palmitic acid can induce inflammatory responses in macrophages, potentially affecting immune memory and responses to pathogenic stimuli (Seufert et al., 2022). Innis (2016) reported that despite its reputation for contributing to chronic disease in adults, palmitic acid is actually a vital component of membrane, secretory, and transport lipids, where it plays a critical role in protein palmitoylation and the production of signal molecules. Linoleic acid is not easily synthesized in the human body and must be obtained from food (Maran & Priya, 2015). It serves as a precursor to omega‐6 fatty acids, which play a crucial role in the functioning of cell membranes and the integrity of the immune system (Kousparou et al., 2023). Furthermore, it has good nutritional effects and health impacts, such as protection against heart disease and anticancer qualities (Carvalho et al., 2011). Omega‐3 fatty acids, specifically alpha‐linolenic acid and stearidonic acid, found in plant oils, are vital for health due to their preventive effects on chronic diseases and their role in cell membranes, inflammation, and cardiovascular health (Yadav et al., 2020). In addition, alpha‐linolenic acid can be oxidized to generate beneficial compounds like phytoprostanes and phytofurans, which have antioxidative and anti‐inflammatory properties (Leung et al., 2022). Furthermore, studies show that alpha‐linolenic acid supplementation in the diet improves lipid profiles, reduces inflammation, and lowers the risk of cardiovascular diseases and mortality (Cambiaggi et al., 2023). Additionally, research has focused on enhancing oilseed crops to accumulate beneficial fatty acids like gamma‐linolenic acid and stearidonic acid, which are important for human health (Lee et al., 2019).

### 3.9 Water‐soluble vitamins

The concentrations of water‐soluble vitamins in *A. abyssinica* and *A. arborescens* plant samples are shown in Table 6. Significant variations (*p* < .05) were observed in the levels of vitamins analyzed in the tested plants. *Artemisia arborescens* exhibited higher levels of vitamin B complex compared to *A. abyssinica*. Among the B‐complex vitamins, thiamin was the most abundant in both plants, with *A. arborescens* showing higher levels (63.63 ± 1.09 mg/100 g DW) than *A. abyssinica* (30.74 ± 1.22 mg/100 g DW). Also, riboflavin, pyridoxine, folic acid, and niacin were also higher in *A. arborescens* (7.39 ± 0.21, 6.07 ± 0.04, 4.66 ± 0.02, and 0.95 ± 0.03 mg/100 g DW, respectively) compared to *A. abyssinica* (1.58 ± 0.01, 4.02 ± 0.06, 3.31 ± 0.01, and 0.52 ± 0.01 mg/100 g DW, respectively). A deficiency in B‐complex vitamins can have detrimental effects on growth and overall health. Thiamin plays a crucial role in various bodily functions, including energy metabolism and nervous system function. So, thiamin deficiency can lead to reduced appetite, slowed growth, weakness, hair loss, and psychological depression (Mohammed et al., 2019). Riboflavin is essential for metabolizing fatty acids, nutrient catabolism in the liver, and maintaining the health of the eyes and skin. Moreover, riboflavin deficiency can contribute to health issues such as cancer and cardiovascular diseases, while pyridoxine is important for combating anemia (Al‐Bukhaiti et al., 2019). So, consuming the *A. arborescens* plant can provide numerous health benefits due to its rich B‐complex vitamin content, supporting various bodily functions and overall well‐being.

**TABLE 6:** Water‐soluble vitamins (mg/100 g DW) of *Artemisia abyssinica* and *Artemisia arborescens* plant powders.

	*A. abyssinica*	*A. arborescens*	RDI (mg)	% C‐RDI	
				*A. abyssinica*	*A. arborescens*
Ascorbic acid [C]	354.52 ± 13.08^a^	4.71 ± 0.03^b^	90 _RDA_	383.91	5.23
Thiamin [B1]	30.74 ± 1.22^b^	63.63 ± 1.09^a^	1.2 _RDA_	2561.67	5302.50
Riboflavin [B2]	1.58 ± 0.01^b^	7.39 ± 0.21^a^	1.3 _RDA_	121.54	568.46
Niacin [B3]	0.52 ± 0.01^b^	0.95 ± 0.03^a^	16 _RDA_	3.25	5.94
Pyridoxine [B6]	4.02 ± 0.06^b^	6.07 ± 0.04^a^	1.3 _RDA_	309.23	466.92
Folic acid [B9]	3.31 ± 0.01^b^	4.66 ± 0.02^a^	–	–	–

*Note*: Different letters show significant changes (*p* < .05) in the same line; values are means ± standard deviation of three determinations.

Regarding vitamin C, *A. abyssinica* had a higher concentration (354.52 ± 13.08 mg/100 g DW) than *A. arborescens* (4.71 ± 0.03 mg/100 g DW). Vitamin C plays a crucial role in enhancing iron absorption in the intestine and is essential for wound healing, tissue repair, collagen formation associated with healthy hair and skin, and immune system support (Ullah & Badshah, 2023). *Artemisia abyssinica*, on the other hand, contained a high level of vitamin C when compared to the levels found by many researchers in citrus fruit, such as Mahdi, Al‐Ansi, et al. (2020), who found 2.39 mg/100 g fresh weight and 0.23 mg/100 g fresh weight in the peel and pulp of foshou fruit, respectively, and higher than reported by Ramful et al. (2010), who registered a value of 34.4–147 mg/100 g fresh weight in peel extract for 21 varieties of citrus fruits, as well as higher than 16 wild plants that had values of 2.80–90.63 mg/100 g DW, reported by Ullah and Badshah (2023). However, meeting the RDI of vitamin C for adults, which is 90 mg/day, can be achieved with 25.39 g of dried *A. abyssinica*, highlighting its potential as a valuable dietary source of this vitamin.

### 3.10 Mono‐ and disaccharides

The mono‐ and disaccharide compositions of *A. abyssinica* and *A. arborescens* plant powders are detailed in Table 1. Both samples contained mono‐ and disaccharides, including glucose and sucrose. Notably, fructose was the predominant monosaccharide in *A. abyssinica*, with a concentration of 602.13 ± 32.12 mg/100 g. In contrast, fructose was not detected in *A. arborescens*. Petropoulos et al. (2019) detected differences in the content of fructose among the samples examined. Their suggestion posits that genotypic variances are the main factor contributing to the variability in individual sugar concentrations, whereas environmental factors and cultivation practices have a lesser impact. Additionally, glucose levels were higher in *A. abyssinica* (58.07 ± 2.41 mg/100 g) compared to *A. arborescens* (42.5 ± 1.08 mg/100 g). Similarly, the content of sucrose was greater in *A. abyssinica* (146.72 ± 8.53 mg/100 g) than in *A. arborescens* (135.1 ± 3.11 mg/100 g). It is worth noting that the levels of mono‐ and disaccharides found in these plants significantly exceed those reported in other plant species such as *C. rotundifolia* leaves (Al‐Bukhaiti et al., 2019), argun palm fruit (Mohammed et al., 2019), foshou fruit (Mahdi, Al‐Ansi, et al., 2020), and various edible wild plants like *Thymus mastichina*, *Pterospartum tridentatum*, *Foeniculum vulgare*, and *Mentha pulegium* (Pinela et al., 2017). The sugar content of a particular plant species is elevated due to multiple factors. Jeandet et al. (2022) discovered a correlation between the level of sunlight exposure and the rate of photosynthesis and sucrose synthesis in plants. Plants that receive ample sunlight contain higher levels of sugar. Also, temperature has an impact on sucrose levels. According to Van den Ende (2014), plants exposed to cooler temperatures contain lower amounts of sugar than plants exposed to warmer temperatures. Moreover, water stress can potentially enhance sucrose accumulation in plant leaves. Zhu et al. (2018) reported that plants experiencing water stress increase their production of sucrose to conserve water. Plant hormones such as auxin and gibberellin can similarly modify the amount of sucrose. Auxin promotes an increase in sucrose levels, whereas gibberellin induces a decrease in sucrose levels (Aluko et al., 2021).

### 3.11 Bioactive compounds

#### 3.11.1 Total phenolic content

**FIGURE 5:**
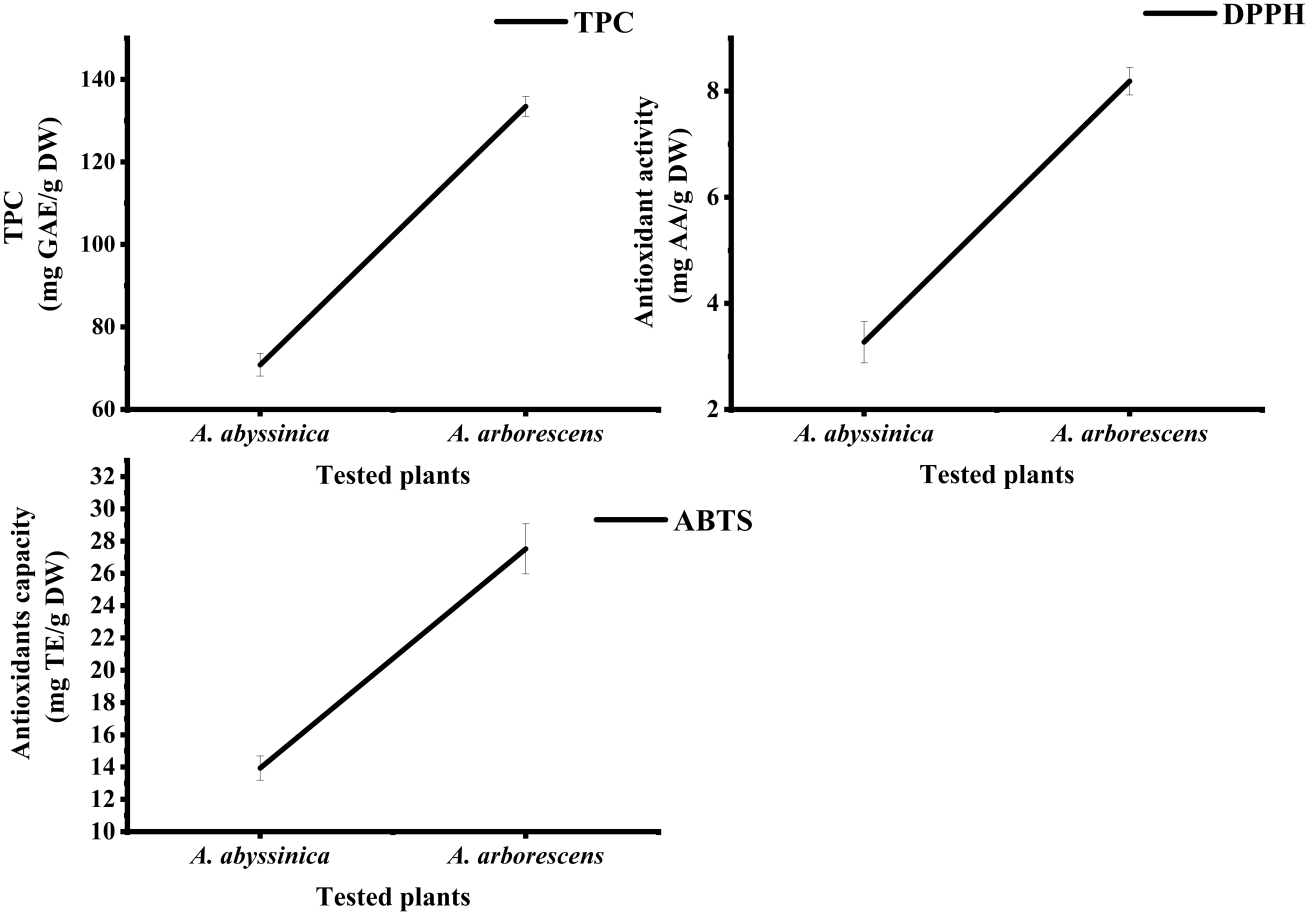
Total phenolic content (TPC), antioxidant activity by DPPH^•^‐SA assay, and antioxidant capacity by ABTS^•+^‐SE assay of *Artemisia abyssinica* and *Artemisia arborescens*.

The TPC of the ethanolic extracts of the evaluated plants is shown in Figure 5. Among the samples, the highest TPC value was observed in the *A. arborescens* extract, measuring 133.43 ± 2.44 mg GAE/g DW. In contrast, the TPC value for the *A. abyssinica* extract was notably lower at 70.85 ± 2.75 mg GAE/g DW, with a statistically significant difference (*p* < .05). These results are consistent with previous findings in supercritical fluid (SFE‐CO2) extracts of *A. abyssinica* (58.95 ± 2.66 mg GAE/g DE) and *A. arborescens* (143.86 ± 9.96 mg GAE/g DE) (Al‐Maqtari, Al‐Ansi, et al., 2021), as well as methanolic extract of *A. arborescens* (110.67 mg GAE/g DE) (Araniti et al., 2016), various extracts of *A. arborescens* and *A. inculta* (Lantzouraki et al., 2023), and micropropagated plants of *A. arborescens* (Riahi et al., 2022). However, it surpasses the levels found by Mayuri et al. (2022) in methanolic extracts of *A. stelleriana* and the ethanolic extract of *A. argentea* reported by Mohammed et al. (2022). These studies suggest that phenolic compounds are the predominant antioxidants, exhibiting scavenging efficiency against reactive oxygen species. Such variations are likely attributed to differences in the extraction methods and the plant species under investigation.

Consumption of foods with high TPC has numerous health advantages. According to Grace (2005), polyphenols, a subclass of phenolic compounds, function as antioxidants, safeguarding cells against oxidative harm induced by free radicals. Consequently, polyphenols mitigate the likelihood of diverse degenerative ailments associated with oxidative stress, possess antibacterial and anti‐inflammatory capabilities, and boost the body's immune system (Wu et al., 2021). Furthermore, these compounds have been linked to enhancing cardiovascular well‐being and exerting a beneficial influence on lipid profiles, blood pressure, insulin resistance, and systemic inflammation (Rana et al., 2022). Additionally, polyphenols like flavan‐3‐ols have been linked to a decreased risk of stroke, myocardial infarction, insulin resistance, and diabetes, as well as improvements in endothelial‐dependent blood flow (Fraga et al., 2019). Therefore, consumption of foods rich in phenolic compounds could be an effective strategy to mitigate noncommunicable diseases and promote overall health.

#### 3.11.2 Antioxidant activity and capacity

The antioxidant properties of plant extracts were assessed using DPPH^•^ and ABTS^•+^‐SE, and the results are presented in Figure 5. Among the two plant extracts, *A. arborescens* extract exhibited the highest antioxidant activity and capacity, measuring 8.19 ± 0.26 mg AA/g DW and 27.52 ± 3.56 mg TE/g DW, respectively. In contrast, *A. abyssinica* showed lower values with 3.27 ± 0.39 mg AA/g DW and 13.94 ± 1.5 mg TE/g DW for antioxidant activity and capacity, respectively. The superiority of *A. arborescens* extract compared with *A. abyssinica* is primarily due to its higher phenolic compounds. As shown in Figure 5, the TPC also supports these findings and explanations. According to Mayuri et al. (2022), these antioxidant properties are often associated with reductones, compounds capable of reducing power and breaking radical chains. So, the higher reducing power in the ethanolic extract corresponds to its superior antioxidant activity. Mohammed et al. (2022) also highlighted that phenolic compounds are the primary antioxidants in the context of their high scavenging ability. This arises from their capacity to neutralize reactive oxygen species, which are free radical compounds. Furthermore, *Artemisia* has antioxidant secondary metabolites, and the solvent type and concentration affected *Artemisia* extract yield and antioxidant activity. Sembiring et al. (2022) stated that ethanol had a higher extract yield and antioxidant activity than methanol, and the IC_50_ of *Artemisia* ethanolic extracts was 20.61 ppm. However, this finding aligns with prior research on *A. abyssinica* (36.97 μg/mL) (Achamo et al., 2022), *A. arborescens* (178 μg/mL) (Dhibi et al., 2016), and *A. annua* (103.247 μg/mL) (Karimi et al., 2023), where phenolic compounds with strong free radical scavenging abilities played a significant role. Nevertheless, it is worth noting that the values obtained in this study were lower than those reported in a previous study by Al‐Maqtari, Al‐Ansi, et al. (2021). These variations may be attributed to differences in the extraction methods employed.

Oxidative/nitrosative stress arises from an equilibrium between free radicals and the body's capacity to neutralize them, leading to a pathological state (Kurutas, 2015). This stress instigates harm to cellular components like proteins, nucleic acids, and lipids, undermining cellular integrity and functionality, thereby fostering the onset of various illnesses (Dutta et al., 2024). Antioxidants serve a crucial role not only in preventing photooxidative processes by functioning as scavengers of oxygen peroxyl radicals and preventing the creation of free radicals, but also in lowering different illnesses such as brain dysfunction, immune system decline, cataracts, cardiovascular disease, hypertension, obesity, aging, and cancer (Alasalvar et al., 2023; Rani & Anisha, 2022). Also, antioxidants boost the immune system by promoting the growth of T lymphocytes in response to infection, increasing the production of cytokines and immunoglobulins, and safeguarding the stability of cell membranes, including the removal of reactive oxygen species (Rani & Anisha, 2022).

## 4 CONCLUSIONS

The comprehensive investigation into the nutritional qualities, functional attributes, and phytochemical compositions of *A. abyssinica* and *A. arborescens* revealed their immense potential for various applications and their promising potential as functional nutritional supplements. Both plants boast favorable nutritional profiles, making them valuable ingredients for diverse food products. *Artemisia abyssinica*'s notable water‐holding capacity positions it as a desirable component for baked goods and other food creations. The presence of unique volatile compounds in both plants also contributes to potential health benefits. Of particular note is the elevated levels of essential amino acids in these plants, which are essential for tissue, enzyme, and hormone synthesis. Moreover, they serve as rich sources of essential minerals like calcium, potassium, magnesium, sodium, and iron, along with vital vitamins such as C and B complex. These nutritional components play crucial roles in providing protective effects against diseases such as cancer and cardiovascular conditions. *Artemisia arborescens*, in particular, stands out for its potent antioxidant properties and high TPC, suggesting substantial health benefits. These findings highlight the untapped potential of *A. abyssinica* and *A. arborescens* in food formulation and product development, offering promising avenues to address nutritional security concerns and promote overall health and well‐being. Further research into the specific mechanisms of action and potential applications of these plant‐derived compounds is warranted to fully harness their therapeutic and functional potential.

## AUTHOR CONTRIBUTIONS

Qais Ali Al‐Maqtari: conceptualization, methodology, software, validation, formal analysis, investigation, and writing – original draft. Jalaleldeen Khaleel Mohamme: conceptualization, methodology, data curation, and review editing. Amer Ali Mahdi: conceptualization, methodology, validation, and formal analysis. Norzila Othman: conceptualization, validation, investigation, data curation, writing – review and editing, visualization, supervision, project administration. Waleed Al‐Ansi: methodology, writing – review and editing. Abeer Essam Noman: writing – review and editing. Adel Ali Saeed Al‐Gheethi: software analysis. Syazwani Mohd Asharuddin: methodology.

## CONFLICT OF INTEREST STATEMENT

The authors declare that they have no conflict of interest.

## ETHICAL APPROVAL

This manuscript did not contain any individual persons' data in any form (including individual details, images, or videos).

## Data Availability

The data that support the findings of this study are available from the corresponding author upon reasonable request.
